# Burnout combating strategies, triggers, implications, and self-coping mechanisms among nurses working in Saudi Arabia: a multicenter, mixed methods study

**DOI:** 10.1186/s12912-025-03191-w

**Published:** 2025-05-26

**Authors:** Mohammad J. Jaber, Alanoud A. Bindahmsh, Omar G. Baker, Amal Alaqlan, Samy M. Almotairi, Ziyad E. Elmohandis, Mahmoud N. Qasem, Hind M. AlTmaizy, Susanna E. du Preez, Raghad A. Alrafidi, Abeer M. Alshodukhi, Faisal N. Al Nami, Baraa M. Abuzir

**Affiliations:** 1 Faculty of Nursing, University of Windsor, Windsor, ON Canada; 2 Department of Nursing, Emergency Center, King Fahad Medical City, Riyadh Second Health Cluster, Riyadh, Saudi Arabia; 3 College of Nursing, King Saud University, Central Region, Riyadh City, Saudi Arabia; 4 Department of Nursing, Emergency Center, King Faisal Specialist Hospital & Research Center, Central Region, Riyadh, Saudi Arabia; 5 Department of Nursing, Cardiac Cath Lab Center, King Saud Medical City, Riyadh First Health Cluster, Central Region, Riyadh, Saudi Arabia; 6 Department of Nursing, Emergency Center, King Khalid University Hospital, Central Region, Riyadh, Saudi Arabia; 7 Department of Nursing, Emergency Center, Dr. Sulaiman Al-Habib Medical Group, Central Region, Riyadh City, Saudi Arabia; 8 Department of Nursing, Education and Practice Improvement Administration, King Fahad Medical City, Riyadh Second Health Cluster, Riyadh, Saudi Arabia; 9 Department of Nursing, Quality Improvement Administration, King Fahad Medical City, Riyadh Second Health Cluster, Riyadh, Saudi Arabia

**Keywords:** Nurse burnout, Coping mechanism, Healthcare workforce, Nursing, Saudi Arabia

## Abstract

**Background:**

The World Health Organization (WHO) has defined burnout as an occupational phenomenon resulting from unsuccessfully managed chronic workplace stress. The well-being of healthcare professionals is the foundation for the health of patients and organizations. In Saudi Arabia, nursing plays a pivotal role in the healthcare sector, with both local and expatriate nurses taking part in various clinical and high-pressure services. However, rapid growth in healthcare facilities, staff shortages, and patient loads are adversely impacting stress levels among nurses.

**Methods:**

This study used a mix of research methods to survey 1,747 nurses (with a 90% response rate) from three major hospitals (King Fahad Medical City, King Saud Medical City, and King Faisal Specialist Hospital and Research Center), two smaller hospitals (Prince Mohammed Bin Abdulaziz Hospital and Al Habib Medical Group (HMG)-Al Suwaidi Hospital branch), and five primary healthcare centers, selecting participants randomly. Survey questionnaires collected data to evaluate the significance of the proposed burnout-combating strategies in relation to nursing administration, workload, and hospital administration responsibilities. Qualitative data were gathered through semi-structured interviews with 90 nurses to investigate and explore burnout triggers, implications, and coping mechanisms.

**Findings:**

A majority of the nurses (87.9%) indicated that assessing their needs and listening to their feedback could help them manage and prevent burnout. In comparison, 89.7% believed that their leaders should enhance the work environment and conditions, while 87% suggested adjusting the nurse-to-patient ratio to improve patient satisfaction. Conversely, 6.8% of the participants held a negative perception that each additional patient per nurse was associated with an increase in the mortality rate, whereas 3.4% felt that granting nurses more control over their schedules and conducting departmental meetings to discuss health could help alleviate work pressure. We identified three themes: factors that trigger burnout symptoms, the implications of burnout, and suggested coping mechanisms. Additionally, they highlighted the prevalence and likelihood of burnout triggers, implications, and coping strategies, providing critical insights for nurse leaders, workload management, and hospital administrators.

**Conclusion:**

Nurse leaders, along with workload management strategies and hospital administrators, play a crucial role in mitigating and overcoming burnout. Establishing a healthy work environment is recognized as the most effective strategy for combating burnout, followed by implementing mental health education and training programs to enhance adaptive and cognitive resilience, promote health improvement, and strengthen resistance to burnout. Further research is needed to evaluate the effectiveness of these coping strategies for other healthcare professionals and to explore how cultural diversity, religious beliefs, and social factors may influence burnout triggers, consequences, and the development of self-coping mechanisms.

**Implications for practice:**

Healthcare leaders should remain vigilant and prioritize strengthening resilience in hospital settings. Changes in institutional policies are essential to upholding suitable staffing ratios to reduce workload stress, implementing equitable scheduling practices to enhance work-life balance, and ensuring consistent, uninterrupted breaks to promote mental and physical rejuvenation. Organizations must implement policies that directly address the factors contributing to burnout.

**Clinical trial number:**

Not applicable.

**Supplementary Information:**

The online version contains supplementary material available at 10.1186/s12912-025-03191-w.

## Introduction

### Background

The World Health Organization (WHO) has defined burnout as an occupational phenomenon that arises from poorly managed chronic workplace stress [1]. The well-being of healthcare professionals serves as the foundation for the health of patients and organizations [2].

Burnout is a psychological and emotional response experienced by workers facing chronic stress [3]. Furthermore, chronic workplace stress is a complex phenomenon that has both physical and emotional consequences for individuals, affects their work with clients and within an organization, and leads to ongoing feelings of emotional exhaustion (EE), depersonalization (DP), and professional inefficacy (PI) [4].

Given that emotional exhaustion, depersonalization, and lack of personal accomplishment act concomitantly to culminate in burnout, this affects job performance, increases medical errors, decreases patient satisfaction, and increases turnover rates. Burnout, therefore, can be described as a syndrome consisting of emotional exhaustion, depersonalization, and reduced personal accomplishment, leading to the inability to perform work-related tasks [5]. One environment that appears very conducive to burnout syndrome is within stressful hospitals and emergency departments that harbor nurses working long hours, complicated cases, extreme emotional strain, etc.

The nursing workforce in Saudi Arabia is crucial in providing healthcare services, and this workforce consists of both Saudi and expatriate nurses working in diverse, difficult clinical settings [6]. The stress levels among nurses are exacerbated, though, because of rapid healthcare facility expansions, shortages in the workforce, and an increase in patient admissions. A study shows that nurses in Saudi Arabia experience a high level of burnout, particularly in critical care units, emergency departments, and general medical wards [7]. Important burnout stressors comprise heavy patient loads, understaffing, lack of administrative support, violence at the workplace, and emotional distress from rendering patient care. Cultural expectations, long shifts, and striated rank orders in the workplace represent additional contributors to the occupational stress that aggravates burnout among nurses in Saudi healthcare settings.

Burnout affects not only the individual nurse but also the healthcare systems in general. Increased absenteeism, increased rates of attrition, and decreased patient care quality have characterized burnout [8]. Chronic stress and depletion may even result in psychopathologies such as anxiety and depression, thus impeding the overall productivity and efficiency of the nursing staff. These factors necessitate that burnout be tackled to ensure the retention of nurses; it also keeps patients safe and improves health in Saudi Arabia’s burgeoning healthcare sector.

Burnout treatment will need some combination of both institutional interventions and self-coping strategies. Institutions can allow and run workload management schemes, psychological support programs, flexible schedules, and opportunities for professional development to advance nurses’ well-being [9]. On the individual level, self-coping mechanisms such as mindfulness and exercise, peer support, or stress management techniques are most often used by nurses to ameliorate burnout symptoms. Unfortunately, their effectiveness depends on individual resilience, workplace culture, and the availability of institutional support.

This multicenter, mixed-methods study intends to explore the triggers, implications, and self-coping mechanisms of burnout among nurses in Saudi Arabia, along with evidence-based recommendations for burnout prevention and management. Getting to know such matters would surely manipulate strategies toward nurses’ wellness, job satisfaction, and sustainable development for the workforce in the health system of Saudi Arabia.

### Literature review

Burnout is an international concern today among nurses, especially in high-stress healthcare environments. It is defined as emotional exhaustion, depersonalization, and reduced personal accomplishment [10]. Burnout in nurses has been studied extensively, as research has shown that it has adverse consequences on the quality of healthcare, the safety of patients, and the well-being of nurses. The present study focuses on the incidence and effect of burnout in nurses, its contributing factors, the role of leadership, coping strategies, and previous studies on nursing burnout in Saudi Arabia.

Healthcare professional burnout is a widespread issue that risks patient safety and is associated with nursing burnout and its repercussions for healthcare systems [11, 12]. Moreover, burnout nurses may experience a sense of failure, physical illness, loss of motivation, or withdrawal from responsibilities [12, 13]. Factors contributing to burnout can be addressed at the interpersonal, individual, and organizational levels [14].

Nurse leaders are crucial in enhancing work conditions, ensuring autonomy, and inspiring nurses to reduce feelings of burnout [15, 16]. Grasping the concept of burnout and recognizing its predisposing factors boosts staff performance and enhances patient care [17, 18]. Therefore, it is critical to recruit more nurses to reduce the workload and adjust working hours to prevent workplace-related burnout [19].

During the COVID-19 pandemic, adequate staffing must be ensured through ongoing workload evaluation, including efforts to reduce workload overtime, avoid long shifts, and deploy staff in areas where training is lacking [20]. An excessive workload increases burnout symptoms and encourages nurses to take sick leave more frequently [21].

To fully address and manage burnout, organizations need to adopt strategies that improve their organizational culture and climate to ameliorate burnout [4]. Burnout is associated with a greater intention to leave the job, low resilience, and supervisory support [22]. Significant correlations were noted between workload and burnout, between burnout and turnover intention, and between workload and turnover intention [23]. The nature of the activity and years of experience were found to have a significant effect on the workload, which, in turn, is associated with levels of burnout [24].

Previous literature shows that the most prevalent and efficient coping strategies are emotional and social support, physical activity, self-care, physical distancing, and emotional separation from work; however, listening to the healthcare provider’s needs and preferences regarding some types of training is essential [25]. The types of professions influenced the relationship between coping techniques and burnout symptoms; however, the implications warrant further discussion for future research and the alleviation of burnout by studying and incorporating various factors, such as gender, years of experience, and so on, when examining the connections between burnout symptoms and coping strategies [26]. Since the beginning of the COVID-19 pandemic, nurses, especially in Saudi Arabia, have experienced increased workloads, significant levels of workplace burnout, and unprecedented turnover [12, 27–29].

### Prevalence and impact of burnout on nurses

Studies have well documented the existence of burnout among nurses, with alarmingly high prevalence rates in most studies worldwide. The WHO has recognized this alarming phenomenon as an occupational phenomenon primarily experienced by healthcare professionals, notably nurses [30]. It has been found that around 30–60% of nurses, with the highest rates from the research conducted respectively in intensive care units, emergency departments, and surgical wards, often have burnout symptoms [31].

Burnout does not affect individual nurses alone; healthcare institutions and patients suffer as well. Nurses who experience burnout are likely to develop symptoms of psychological distress, depression, anxiety, and physical exhaustion [32]. Burnout is also associated with absenteeism, lack of job satisfaction, lost productivity, and high chances of committing medical errors [33]. Other studies have indicated a direct relationship between nurses’ burnout and adverse patient outcomes, which include higher infection rates, medication errors, and dissatisfaction among patients [34]. This has put serious challenges to the sustainability of the nursing workforce in Saudi Arabia because of increased nurse turnover attributed to burnout, which results in a shortage of nurses and increased workloads on the remaining nursing staff [35].

### Contributing factors to nurse burnout

Nurse burnout is largely attributed to workplace stressors, organizational dilemmas, and personal issues. Studies have identified a heavy workload, followed by long shift hours and the presence of staffing scarcity, as the prime triggers for burnout [36]. Nurses often work for 12 or more hours at a stretch, which wears them down physically and mentally. Chronic stress and job strain arise from the nature of nursing, especially when care is provided in critical care settings.

Further aggravating burnout are organizational factors like administrative neglect, poor resources, and a lousy work-life balance [37]. According to Kim et al. [38], additional stressors that impinge on job dissatisfaction and intent to leave the profession include bullying and violence in the workplace, a toxic environment, and role ambiguity. Being a country where cultural expectations and hierarchies prevail, Saudi Arabia may even further contribute to work-related stress for expatriates facing language barriers, cultural differences, and job insecurity.

### Role of leadership

This policy will raise the morale of nurses, reduce their burnout, and create working competition among them. The study revealed that transformational leadership was closely associated with lower burnout and higher satisfaction [39]. Therefore, as much as it is a necessary drug transformation, it is so in creating a strong supportive cognitive work environment, or a nurse-led, enhancing psychological support, and better employee well-being programs towards effective reduction of stressors and appeals in improving nurse retention [40].

Research shows the impacts of authentic and servant leadership behaviors: they tend to present open communication and trust in order to realize shared decisions among those involved, and they often lead to a better working environment and reduce emotional exhaustion [41]. Nurse leadership in Saudi Arabia is highly significant in providing support towards workload management and conflict resolution and potential promotion in professional development; however, studies indicate that hierarchical leadership structures endanger such support systems and result in more stress and burnout [42].

### Coping strategies

Nurses engage in various coping strategies to counteract their experience of burnout, from self-directed individual coping strategies to organizational intervention strategies. Although self-coping strategies include mindfulness, relaxation techniques, exercises, and social support [43], nurses also indicated benefits in reducing symptoms of burnout when they underwent peer support, counseling, or stress management workshops [44].

Organizations where workloads are adjusted, more flexible scheduling is considered, and mental health resources are available, experience reduced stress and increased satisfaction [45]. Organizations that integrated burnout prevention programs, trained their leaders in resilience, and invested in well-being were rewarded with less nurse turnover and improved outcomes in health care. In Saudi Arabia, some hospitals have started resilience-enhancing programs, wellness programs, and workshops on professional development to promote nurses’ mental health and job satisfaction [46].

### Previous studies on nursing burnout in Saudi Arabia

Research on nurse burnout has shown that nurses in Saudi Arabia face unique challenges in that country’s healthcare system. For instance, Alqahtani et al. [7] showed that nearly 50% of nurses working in tertiary hospitals experience high levels of emotional exhaustion and depersonalization caused by burnout, with the condition being worse for expatriate nurses. Similarly, Al Sabei and Labrague [47] identified workload, nurse-to-patient ratios, and job dissatisfaction as causes of burnout in nurses in Saudi Arabia. Alhamdan et al. [48] emphasized in their review that effective leadership styles impact burnout. Nurse managers who provide strong support and professional development opportunities help nurses become less stressed and improve retention. Aljohani et al. [46] studied coping strategies used by Saudi nurses and found that self-care, peer support, and religious coping mechanisms helped nurses become more resilient to burnout-associated stressors. Research has indicated that nurse burnout is very much experienced in unique ways by nurses.

The study aimed to investigate the triggers, implications, and coping mechanisms of burnout to alleviate it on personal, individual, and organizational levels. Thus, it sought to assess the significance of strategies for combating nursing administration responsibilities, workload, and hospital administration tasks in various healthcare settings.

The literature highlights the high prevalence and detrimental impact of burnout among nurses, particularly in high-pressure environments. Contributing factors include heavy workloads, poor staffing, lack of support, and stressful work conditions. Effective leadership, coping strategies, and institutional interventions play a crucial role in reducing burnout and improving nurse well-being. Studies from Saudi Arabia emphasize the importance of workplace support, resilience-building initiatives, and leadership involvement in combating burnout among nurses. Addressing these issues is essential for ensuring a sustainable nursing workforce and high-quality patient care in Saudi Arabia’s healthcare system.

## Methods

### Study design

Although burnout is a well-documented phenomenon in healthcare settings, the number of healthcare providers, particularly nurses in Saudi Arabia, is rising as many leave or change jobs, especially during and after the pandemic. Therefore, a mixed-methods design was implemented to enhance understanding and gain in-depth knowledge by utilizing triangulation, providing a holistic view of trends and patterns in the quantitative data, as well as the meaning and context behind those patterns in the qualitative data. This approach aimed to increase the validity and credibility of the findings by employing multiple sources of data (both qualitative and quantitative) [49].

## Study settings and procedures

### Survey questionnaires

A total of 1747 participants were surveyed to evaluate the significance of burnout prevention strategies in relation to nursing administration responsibilities (NR), workload (WL), and hospital administration responsibilities (HR) at the following centers:

- Three tertiary hospitals serve the central region of the kingdom: King Fahad Medical City (KFMC) is a 1, 200- bed government hospital that includes four facilities: general, children’s specialized, women’s specialized, and rehabilitation hospitals, along with seven specialized centers: ambulatory, neuroscience, heart, oncology, obesity, endocrine, and metabolism. It offers complex tertiary healthcare services to the public. King Saud Medical City (KSMC) is a 1,400-bed government hospital that encompasses general, pediatrics, and maternity hospitals, as well as a dental center and the King Fahad Charity Kidney Center. It also provides complex tertiary healthcare services to the public. King Faisal Specialist Hospital and Research Center (KFSHRC) is a 1, 519- bed general (royal) hospital that delivers specialized healthcare services through five centers of excellence: an organ transplant center, an oncology center, a genomic medicine center, a neuroscience center, and a heart center.
- One university hospital, King Khalid University Hospital (KKUH), is a research and educational hospital that opened in 1982 at King Saud University Medical City (KSUMC). It has 1,200 beds and provides a full range of general and subspecialty medical and surgical services.
- Two secondary hospitals: Prince Mohammed Bin Abdulaziz Hospital (PMAH) is a 5500-bed government facility that offers secondary healthcare services to the public. Al Habib Medical Group (HMG)-Al Suwaidi Hospital branch is a 305-bed private hospital providing healthcare services across all medical specialties.
- Five primary healthcare centers (PHC) have government facilities, each including primary clinics (pediatric, maternity, internal medicine, and first aid), a primary assessment room, a radiology room, and a pharmacy. These centers provide primary care to the surrounding neighborhood.

## Semistructured interviews

A 30-minute individual semistructured interview intends to gather open-ended data, investigate participants’ thoughts, feelings, and beliefs regarding a specific topic, and thoroughly examine personal and occasionally sensitive matters [50]. The site investigators at KFMC, KSMC, KFSHRC, KKUH, and HMG interviewed 90 nurses to investigate and explore the triggers, implications, and coping mechanisms of burnout.

### Sampling technique

A total of 13,065 nurses were the target population (N) in all participating hospitals. The sample size (*n* = 1,932) was calculated using “Raosoft software” for a cross-sectional study. Calculated sample sizes were as follows: KFMC (*N* = 2500, *n* = 334), KSMC (*N* = 4900, *n* = 357), KFSHRC (*N* = 3000, *n* = 341), KKUH (*N* = 1800, *n* = 317), and PMAH (*N* = 500, *n* = 218), HMG (*N* = 3000, *n* = 340), and PHCs (*N* = 25, *n* = 25). Only 1,747 nurses responded; the response rate was 90%. A total of 90 semi-structured interviews were conducted.

### Data collection

The participants were informed through formal communication (email). The email addresses included the IRB for data collection, an electronic survey (link), and instructions about the study. However, qualitative data were collected through semistructured interviews conducted by site investigators at KFMC, KSMC, KFSPRC, KKUH, and HMG.

### Instrument Preparation

The questionnaire and the burnout combating strategies scale were developed specifically for this study by the study’s authors and were not published elsewhere (Supplemental File 1).

The tool was then thoroughly examined, revised, and assessed by a panel of nursing experts for its face and content validity. A Spearman’s correlational analysis was conducted, yielding a total score for each item that exceeded the r-table product-moment value. According to Spearman’s correlational analysis, *d*f = 1745, *p* < 0.001, and the average total score of 0.556, which is greater than the 0.350 r-table product-moment value, confirming the tool’s validity (Table S1).

Additionally, construct validity was evaluated through exploratory factor analysis utilizing principal component analysis (EFA-PCA) with the Oblimin rotation method (*n* = 1,747) and a cut-off loading factor of 0.60. The results were deemed appropriate based on the Kaiser-Meyer-Olkin (KMO) measure of sampling adequacy and Bartlett’s Test of Sphericity (0.965), (*p* < 0.001), and *d*f (630) (Table S2).

A small exploratory (pilot) analysis of the tool (36 questions) was conducted on 32 participants to assess the reliability (statistical consistency) in different settings and units, revealing excellent reliability (Cronbach’s alpha = 0.937) (Table S3).

The qualitative semistructured survey items were collated from the literature and the experiences of senior nurses to dig deeply into the triggering factors and implications of burnout at individual, personal, and organizational levels and explore their self-coping and adaptive mechanisms. The semistructured interview checklist (3 themes) was tested on 5 participants for clarity, language, time, and comprehension to ensure an in-depth understanding of burnout triggers, implications, and coping mechanisms.

### Measurement scales

#### Burnout combating strategies scale

This scale uses a 3-point Likert scale measurement and aims to assess the roles of nursing administration, the impact of workload, and hospital administration to overcome burnout.

#### Burnout triggers, implications, and coping mechanisms

The qualitative section explores the participants’ deep feelings and perceptions about the knowledge of burnout triggers, implications, and coping mechanisms.

### Data analysis

The quantitative data were analyzed using IBM SPSS version 27. A normality test was performed, and the data were not normally distributed (*p* ≤ 0.001); therefore, nonparametric analyses were used using the mean rank (median) and interquartile range (IQR) for describing values and their frequencies, and Mann-Whitney and Kruskal-Wallis analyses for comparing the participant characteristics and work conditions. In addition, predictive analysis using a Spearman correlation coefficient was used to predict the associations between the outcome variables and the explanatory (independent) variables. All hypotheses were tested for the 2-tailed significance level, *P* ≤ 0.050, and 95% confidence intervals (Table S4).

The qualitative data were analyzed using the broad principles of thematic analysis to identify themes relating to perceived knowledge of burnout triggers, implications, and alleviating coping mechanisms.

### Findings

#### Participant characteristics

A total of 1,747 participants were recruited from multi-center (governmental, general, private, educational, and public centers) settings. The majority of participants were females (83%), aged 31 to 35 years (27.9%), married (52.4%), Asian (50.6%), had a bachelor’s degree (74.7%), worked in direct care (72.2%), and RN (84.8%) (Table S5).

#### Participant work conditions

The majority of participants worked in a tertiary (63.7%) governmental sector (58.6%), worked in an inpatient setting (36.2%), performed a 12-hr rotating shift (48.5%), worked 46–48 h per week (47.9%), used a total patient care model (73.3%), experienced democratic leadership (53.3%) with 6–10 years of nursing experience (25.6%), and had 1–5 years of hospital experience (33.1%). Concerning the previous experience of burnout, 63.4% had previous experience, 76.8% did not receive training on burnout, and 84.7% received proper orientation upon arrival at their unit (Table S6).

#### Participant responses

Table S7 shows the nurses’ positive and negative responses regarding nursing administration responsibilities, the impacts of workload, and hospital administration duties.

#### Nursing administration responsibilities (NR)

**Fig. 1:**
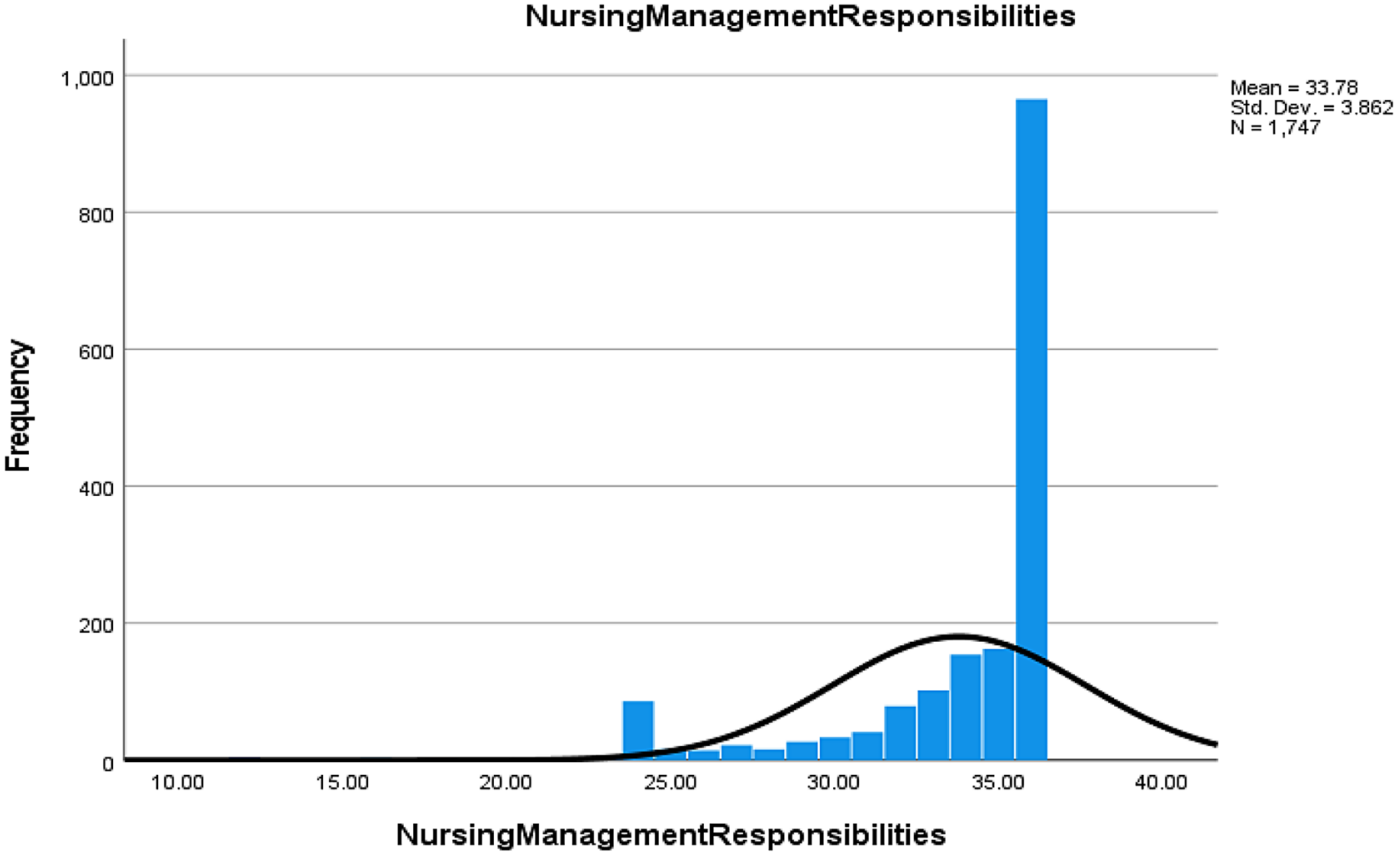
Nurse responses regarding the roles of nurse leaders in detecting and managing burnout

87.9% of nurses indicated that assessing their needs and listening to their feedback could help them manage and prevent burnout (Table S7, Fig. 1).

#### Impact of workload (WL)

**Fig. 2:**
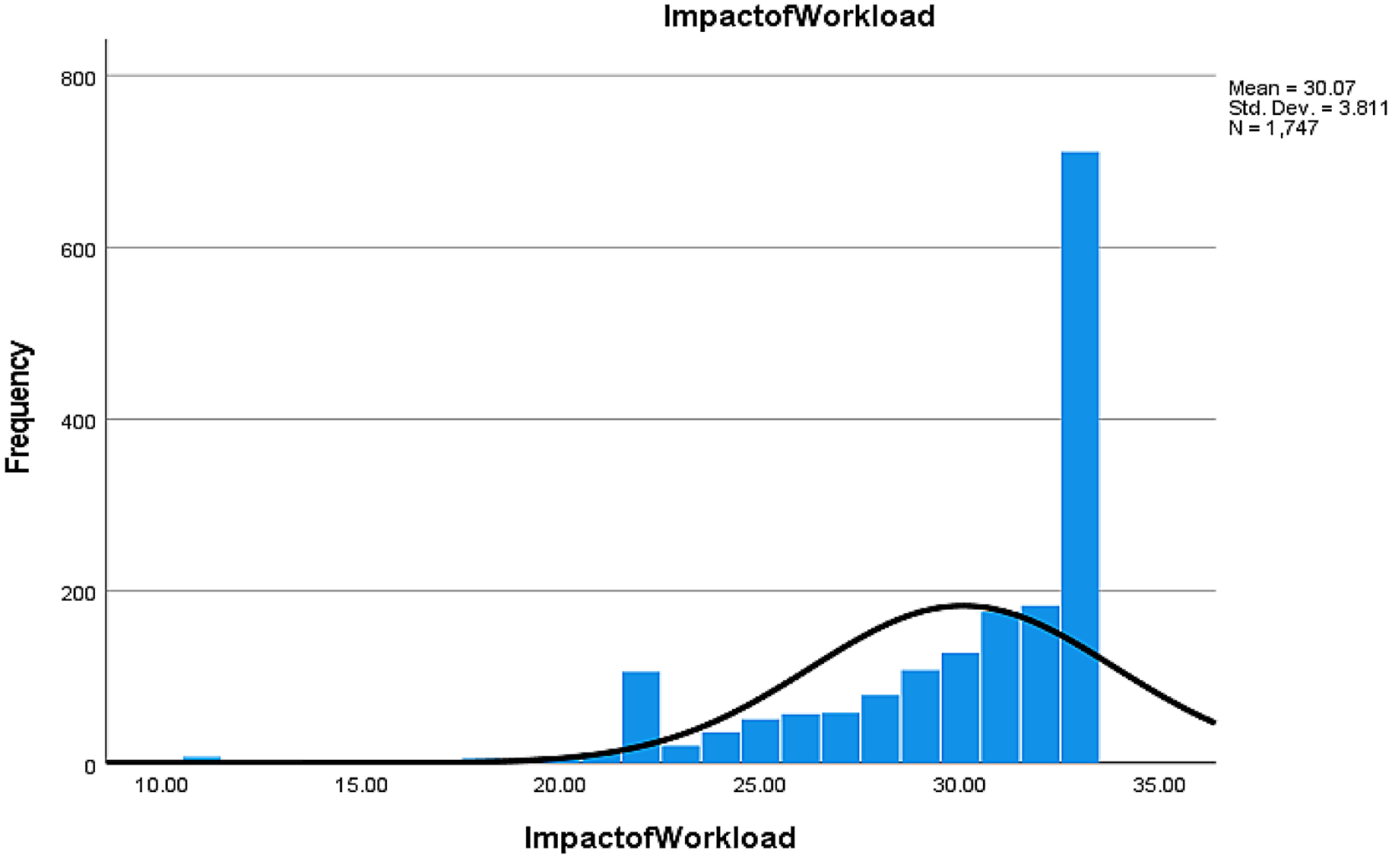
Nurse responses regarding the impact of workload in triggering burnout

89.7% of nurses believed that their leaders should enhance the work environment and conditions, and 87% suggested adjusting the nurse-to-patient ratio to improve patient satisfaction (Table S7, Fig. 2).

#### Hospital administration responsibilities (HR)

**Fig. 3:**
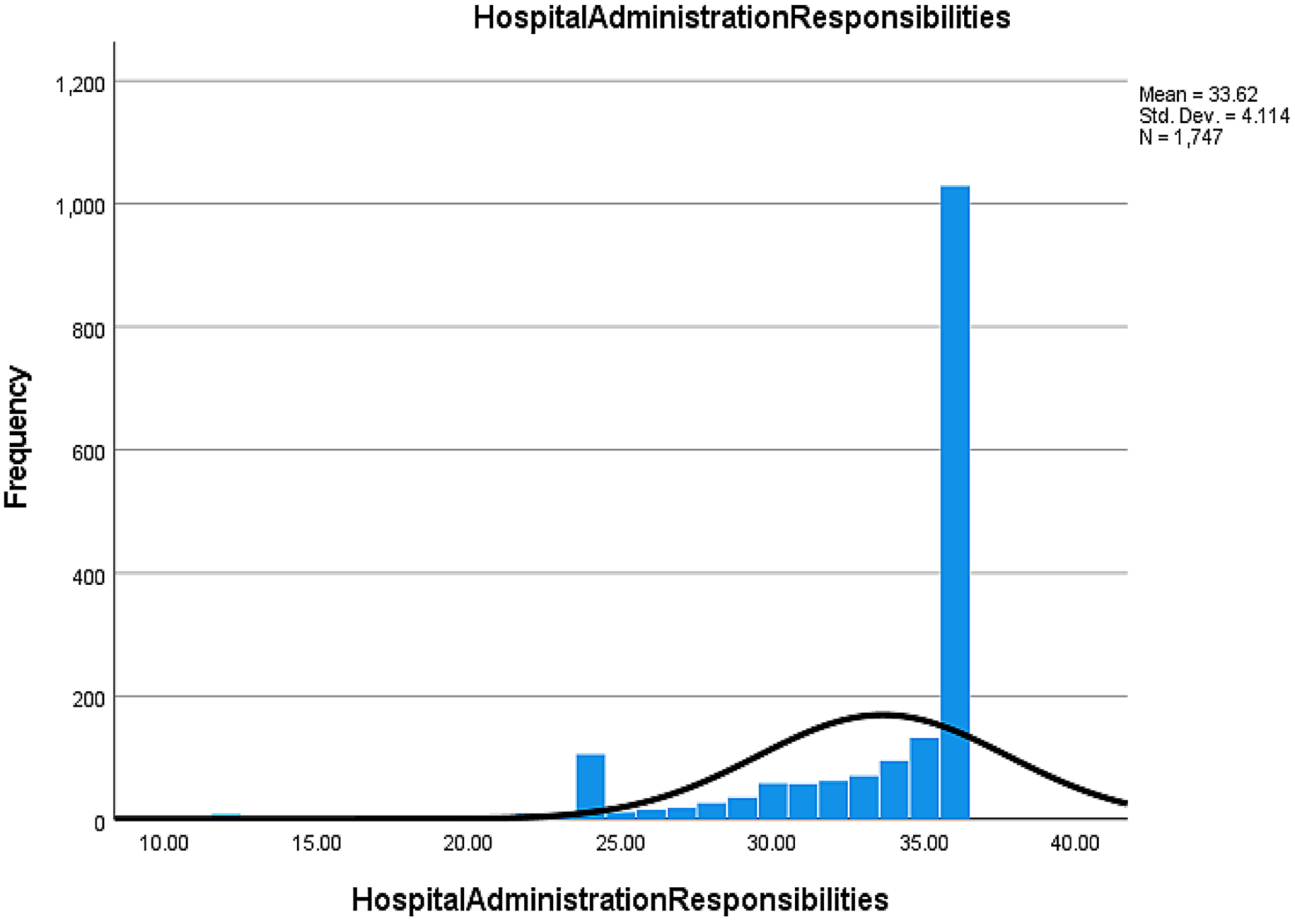
Nurse responses regarding the roles of nurse leaders managing burnout

**87.9%** of nurses believed that assessing their needs and listening to their feedback by the hospital administrators helps them cope and prevent burnout (Table S7, Fig. 3).

### Comparing participant responses

Table 1 shows a significant difference in addressing burnout combating strategies between those who experienced burnout and those who did not, particularly regarding the importance of nurse leader roles, workload management strategies, and hospital administrator roles (*p* < 0.001) across all domains. Furthermore, there was a notable difference between participants who received orientation upon arrival at the hospital and those who did not (*p* = 0.012) concerning the roles of hospital administrators in combating burnout triggers.

**Table 1:** Mann-Whitney analysis of dichotomous variables (*n* = 1747)

Outcome Variables	Independent Variables	*n*	Mean Rank	Z	Mann-Whitney U	Asymp. Sig.* P value
	***Previous experience of work-related burnout***					
Nursing Administration Responsibilities	Yes	1108	927.49	-6.408	294738.5	**< 0.001**
	No	639	781.25			
Impact of Workload	Yes	1108	913.15	-4.431	310622.5	**< 0.001**
	No	639	806.11			
Hospital Administration Responsibilities	Yes	1108	916.26	-5.172	307,182	**< 0.001**
	No	639	800.72			
	***Proper orientation upon arrival at the unit***					
Nursing Administration Responsibilities	Yes	1479	880.33	-1.352	188,829	0.176
	No	268	839.09			
Impact of Workload	Yes	1479	871.47	-0.511	194,442	0.609
	No	268	887.97			
Hospital Administration Responsibilities	Yes	1479	885.51	-2.513	181,167	**0.012**
	No	268	810.5			

*Statistically significant at (α ≤ 0.050)

Table 2 highlights significant differences among the participants’ responses to burnout-combating strategies across the following variables: age in relation to the role of nurse leaders (*p* = 0.001), workload management strategies (*p* = 0.009), and the role of hospital administrators (*p* < 0.001); ethnicity concerning workload management strategies (*p* = 0.001) and the role of hospital administrators (*p* < 0.001); type of hospital relating to both the role of nurse leaders and workload management strategies (*p* < 0.001); hospital level in terms of workload management strategies (*p* = 0.001); type of work regarding the role of hospital administrators (*p* = 0.011); type of shift relating to the role of nurse leaders (*p* = 0.034) and workload management strategies (*p* = 0.023); leadership style concerning the role of hospital administrators (*p* = 0.027); years of nursing experience related to both the role of nurse leaders and hospital administrators (*p* < 0.001); and years of hospital experience pertaining to the role of nurse leaders (*p* = 0.045) and the role of hospital administrators (*p* < 0.001).

**Table 2:** Kruskal-Wallis analysis of continuous variables (*n* = 1747)

Outcome Variables	Independent Variables	*n*	Mean Rank	df	Kruskal-Wallis H	Asymp. Sig.* P value
	***Age***					
Nursing Administration Responsibilities	≤ 25	191	791.21	4	18.07	**0.001**
	26–30	418	833.58			
	31–35	487	930.35			
	36–40	352	864.63			
	>40	299	902.64			
Impact of Workload	≤ 25	191	821.84	4	13.60	**0.009**
	26–30	418	837.2			
	31–35	487	918.81			
	36–40	352	917.87			
	>40	299	834.14			
Hospital Administration Responsibilities	≤ 25	191	808.65	4	18.66	**< 0.001**
	26–30	418	819.11			
	31–35	487	899.26			
	36–40	352	936.68			
	>40	299	877.55			
	Widow	12	1036.17			
	Divorced	30	965.03			
	Separated	22	929.61			
	***Ethnicity/Race***					
Nursing Administration Responsibilities	Middle East	406	867	6	7.88	0.247
	Asia	884	890.73			
	Africa	78	931.08			
	America	4	1124			
	Europe	36	864.32			
	Hispanic/Latino	158	813.34			
	India	181	832.78			
Impact of Workload	Middle East	406	826.74	6	21.73	**0.001**
	Asia	884	913.08			
	Africa	78	762.91			
	America	4	806.63			
	Europe	36	895.81			
	Hispanic/Latino	158	929.22			
	India	181	785.96			
Hospital Administration Responsibilities	Middle East	406	798.49	6	29.85	**< 0.001**
	Asia	884	929.69			
	Africa	78	859.16			
	America	4	767.63			
	Europe	36	768.21			
	Hispanic/Latino	158	837.34			
	India	181	833.17			
	***Type of hospital***					
Nursing Administration Responsibilities	Governmental	1023	887.53	4	38.99	**< 0.001**
	General	141	931.99			
	Educational	94	1038.55			
	Private	462	781.32			
	Public Health Centers	27	1071.69			
Impact of Workload	Governmental	1023	904.49	4	23.08	**< 0.001**
	General	141	853.73			
	Educational	94	964.34			
	Private	462	788.27			
	Public Health Centers	27	977.15			
Hospital Administration Responsibilities	Governmental	1023	877.63	4	9.44	0.051
	General	141	909.13			
	Educational	94	954.25			
	Private	462	832.53			
	Public Health Centers	27	983.2			
	***Level of hospital***					
Nursing Administration Responsibilities	Tertiary	1112	878.48	3	4.33	0.228
	Secondary	341	892.39			
	Primary	252	849.72			
	Clinic	42	751.7			
Impact of Workload	Tertiary	1112	870.5	3	15.58	**0.001**
	Secondary	341	941.01			
	Primary	252	832.29			
	Clinic	42	672.74			
Hospital Administration Responsibilities	Tertiary	1112	885.69	3	6.44	0.092
	Secondary	341	881.89			
	Primary	252	833.51			
	Clinic	42	743.5			
	***Type of work***					
Nursing Administration Responsibilities	Administrative	70	761.99	4	6.96	0.138
	Clinical	1262	877.86			
	Education	10	963.2			
	Quality	8	1122.31			
	Other	397	874.24			
Impact of Workload	Administrative	70	840.39	4	0.44	0.979
	Clinical	1262	875.15			
	Education	10	843			
	Quality	8	843.94			
	Other	397	877.66			
Hospital Administration Responsibilities	Administrative	70	708.74	4	12.97	**0.011**
	Clinical	1262	879.85			
	Education	10	1036.45			
	Quality	8	662.38			
	Other	397	884.73			
	***Type of shift***					
Nursing Administration Responsibilities	Morning (8-9-hr)	359	880.05	4	10.44	**0.034**
	Day (12-hr)	259	856.9			
	Night (12-hr)	84	965.61			
	Rotating shift (12-hr)	847	886.38			
	Rotating shift (8-9-hr)	198	793.6			
Impact of Workload	Morning (8-9-hr)	359	867.77	4	11.38	**0.023**
	Day (12-hr)	259	900.77			
	Night (12-hr)	84	892.29			
	Rotating shift (12-hr)	847	891.34			
	Rotating shift (8-9-hr)	198	768.35			
Hospital Administration Responsibilities	Morning (8-9-hr)	359	887.25	4	3.69	0.450
	Day (12-hr)	259	876.43			
	Night (12-hr)	84	930.49			
	Rotating shift (12-hr)	847	872.69			
	Rotating shift (8-9-hr)	198	828.41			
	***Leadership style***					
Nursing Administration Responsibilities	Autocratic	377	847.29	4	3.39	0.494
	Democratic	930	872.69			
	Transactional	133	877.22			
	Transformational	243	916.54			
	Permissive/Laissez-faire	64	882.23			
Impact of Workload	Autocratic	377	842.03	4	2.19	0.700
	Democratic	930	885.29			
	Transactional	133	875.13			
	Transformational	243	881.24			
	Permissive/Laissez-faire	64	868.48			
Hospital Administration Responsibilities	Autocratic	377	841.39	4	10.95	**0.027**
	Democratic	930	868.69			
	Transactional	133	905.89			
	Transformational	243	947.08			
	Permissive/Laissez-faire	64	799.53			
	***Years of nursing experience***					
Nursing Administration Responsibilities	< 1	113	730.11	4	22.75	**< 0.001**
	1‒5	424	823.96			
	6‒10	448	891.85			
	11‒15	381	925.18			
	> 15	381	900.19			
Impact of Workload	< 1	113	768.19	4	8.03	0.090
	1‒5	424	853.88			
	6‒10	448	880.68			
	11‒15	381	904.32			
	> 15	381	889.6			
Hospital Administration Responsibilities	< 1	113	734.9	4	26.13	**< 0.001**
	1‒5	424	823.71			
	6‒10	448	871.16			
	11‒15	381	913.13			
	> 15	381	935.43			
	***Years of hospital experience***					
Nursing Administration Responsibilities	< 1	277	821.33	4	9.76	**0.045**
	1‒5	579	851.62			
	6‒10	392	919.28			
	11‒15	264	897.01			
	> 15	235	889.85			
Impact of Workload	< 1	277	826.47	4	5.27	0.261
	1‒5	579	862.43			
	6‒10	392	901.67			
	11‒15	264	903.99			
	> 15	235	878.69			
Hospital Administration Responsibilities	< 1	277	810.95	4	19.23	**< 0.001**
	1‒5	579	840.72			
	6‒10	392	886.29			
	11‒15	264	947.09			
	> 15	235	927.7			

*Statistically significant at (α ≤ 0.050)

### Predicting relationships among variables

Tables 3 and 4 present the results of multiple regression analysis for predicting the outcome variables based on the explanatory variables related to burnout. The participant’s years of nursing experience (*p* < 0.001), prior burnout experience (*p* = 0.001), and adequate orientation (*p* = 0.014) were identified as significant predictors for nurse leaders in recognizing, addressing, and managing burnout. The type of hospital (*p* = 0.003), years of nursing experience (*p* = 0.015), prior burnout experience (*p* < 0.001), and training in managing burnout (*p* = 0.014) were identified as significant factors influencing workload strategies to combat burnout. Age (*p* = 0.021), weekly working hours (*p* = 0.027), years of nursing experience (*p* = 0.001), prior burnout experience (*p* = 0.015), and adequate orientation (*p* < 0.001) were identified as significant predictors for hospital leaders to implement policies and support programs aimed at managing burnout.

**Table 3:** Multiple regression analysis of participants’ characteristics (*n* = 1747)

Dependent Variables				df		*R* ^2^		F	Sig* (*p*-value)
	Nursing administration responsibilities			19	1726	0.038		3.600	<** 0.001**
	Workload					0.033		3.103	**< 0.001**
	Hospital administration responsibilities					0.035		3.334	**< 0.001**
**Independent** **Variables**		**Dependent** **Variables**	**Beta Coefficient** **(Unstandardized β)**				***t***		
Age		Nursing administration responsibilities	− 0.203				-1.510		0.131
		Workload	− 0.170				-1.275		0.202
		Hospital administration responsibilities	− 0.330				-2.302		**0.021**
Years of nursing Experience		Nursing administration responsibilities	0.565				3.726		**< 0.001**
		Workload	0.367				2.441		**0.015**
		Hospital administration responsibilities	0.516				3.186		**0.001**

*Statistically significant at (α ≤ 0.050)

Legends: Reg-Regression, Resid-Residual

**Table 4:** Multiple regression analysis of participants’ work conditions (*n* = 1747)

Dependent Variables				df		*R* ^2^		F	Sig^*^ (*p*-value)
	Nursing administration responsibilities			19	1726	0.038		3.600	< **0.001**
	Workload					0.033		3.103	**< 0.001**
	Hospital administration responsibilities					0.035		3.334	**< 0.001**
**Independent** **Variables**		**Dependent** **Variables**	**Beta Coefficient** **(Unstandardized β)**				***t***		
Type of hospital		Nursing administration responsibilities	− 0.124				-1.672		0.095
		Workload	− 0.214				-2.929		**0.003**
		Hospital administration responsibilities	− 0.092				-1.163		0.245
Weekly working hours		Nursing administration responsibilities	0.136				0.943		0.346
		Workload	0.161				1.126		0.260
		Hospital administration responsibilities	0.340				2.207		**0.027**
Previous experience of burnout		Nursing administration responsibilities	− 0.671				-3.224		**0.001**
		Workload	− 0.863				-4.191		**< 0.001**
		Hospital administration responsibilities	− 0.539				-2.431		**0.015**
Receiving training on managing burnout		Nursing administration responsibilities	0.288				1.296		0.195
		Workload	0.539				2.448		**0.014**
		Hospital administration responsibilities	0.317				1.334		0.183
Receiving proper orientation upon arrival to the unit		Nursing administration responsibilities	− 0.642				-2.465		**0.014**
		Workload	− 0.103				− 0.399		0.690
		Hospital administration responsibilities	-1.001				-3.606		**< 0.001**

*Statistically significant at α ≤ 0.050

Legends: Reg-Regression, Resid-Residual

Table 5 presents the results of the multivariate analysis predicting the outcome variables based on the independent variables concerning burnout combating strategies. The participant’s professional titles (*p* = 0.004), care delivery method (*p* = 0.011), leadership style (*p* = 0.014), previous burnout experience (*p* = 0.001), training in managing burnout (*p* = 0.014), and proper orientation (*p* = 0.001) were significantly associated with the roles of nurse leaders and hospital administrators and workload management strategies.

**Table 5:** Multivariate and between-subjects effects analysis (*n* = 1747)

		Wilks’ Lambda^a^	Hypo. df	Error df	F	Sig.^b^	Part.^c^ η^2^	Obser. power
**Independent** **Variables**	Professional title	0.972	24.000	4818.007	1.954	**0.004**	0.009	0.994
	Nursing care delivery method	0.987	9.000	3981.745	2.378	**0.011**	0.004	0.847
	Leadership style	0.985	12.000	4328.741	2.105	**0.014**	0.005	0.906
	Previous experience of burnout	0.990	3.000	1636.000	5.332	**0.001**	0.010	0.933
	Receiving training on managing burnout	0.994	3.000	1636.000	3.537	**0.014**	0.006	0.787
	Receiving proper orientation upon arrival to the unit	0.990	3.000	1636.000	5.430	**0.001**	0.010	0.938
**Independent** **Variables**	**Outcome** **Variables**	**Sum of Squares**	***d*** **f**	**Mean Square**				
Professional title	*Nursing administration responsibilities*	210.39	8	26.299	1.78	0.077	0.008	0.771
	*Workload*	176.078	8	22.01	1.529	0.142	0.007	0.693
	*Hospital administration responsibilities*	315.703	8	39.463	2.391	**0.015**	0.011	0.899
Nursing care delivery method	*Nursing administration responsibilities*	168.804	3.000	56.268	3.951	**0.008**	0.007	0.834
	*Workload*	201.355	3.000	67.118	4.775	**0.003**	0.009	0.903
	*Hospital administration responsibilities*	190.939	3.000	63.646	3.911	**0.009**	0.007	0.830
Leadership style	*Nursing administration responsibilities*	56.341	4.000	14.085	0.989	0.412	0.002	0.316
	*Workload*	89.796	4.000	22.449	1.597	0.172	0.004	0.497
	*Hospital administration responsibilities*	314.967	4.000	78.742	4.838	**< 0.001**	0.012	0.958
Previous experience of burnout	*Nursing administration responsibilities*	79.659	1.000	79.659	5.593	**0.018**	0.003	0.657
	*Workload*	197.205	1.000	197.205	14.031	**< 0.001**	0.008	0.963
	*Hospital administration responsibilities*	197.412	1.000	197.412	12.129	**< 0.001**	0.007	0.936
Receiving training on managing burnout	*Nursing administration responsibilities*	23.554	1.000	23.554	1.654	0.199	0.001	0.251
	*Workload*	96.964	1.000	96.964	6.899	**0.009**	0.004	0.747
	*Hospital administration responsibilities*	138.453	1.000	138.453	8.507	**0.004**	0.005	0.830
Receiving proper orientation upon arrival to the unit	*Nursing administration responsibilities*	165.679	1.000	165.679	11.634	**< 0.001**	0.007	0.926
	*Workload*	15.586	1.000	15.586	1.109	0.292	0.001	0.183
	*Hospital administration responsibilities*	125.849	1.000	125.849	7.732	**0.005**	0.005	0.794

Wilks’ Lambdaa value (0.000–1.000): (close to 0.000) means the independent variable does not significantly affect the dependent variables, (close to 1.000) means the independent variable significantly affects the dependent variables

Sig.: Statistically significant at (α ≤ 0.050)

Part.*η*^*2*^: Partial eta squared: low effect ( 0.01–<0.06), medium effect (0.06–<0.14), high effect (≥ 0.14)

### Thematic analysis

Participants expressed their concerns about their well-being, anticipated burnout, and discussed coping mechanisms. The thematic analysis identified three themes. These themes were analyzed as follows:

#### Theme 1–Contributing factors triggering burnout symptoms

This thematic construct evaluated individual nurses’ knowledge in identifying factors that trigger burnout at:

##### Organizational level

Participants highlighted numerous challenges in their work environment. One recurring concern was the impact of “*reduced days off*,” “*workload*,” *and* “*high equity vs.*. *staffing*.” The COVID-19 lockdown is often a persistent issue, as noted by one participant: “*No vacation due to COVID-19 lockdown.*” Participants also expressed concerns regarding “*working hours*,” “*lack of staff*,” *and* “*inadequate compensation*,” which contributed to frustration and dissatisfaction among them. Additionally, the prevailing sense of “*lack of appreciation*” *and* “*low salary*” added to the discontent, along with the “*lack of support system*” *and* “*dysfunctional workplace dynamics.*” The absence of a support system compounded these challenges, with another participant stating, “*delay of a promotion*” *and* “*lack of appreciation.*” One participant remarked: “*Remove financial rewards*,” further exacerbating the situation. “*Unrealistic workload*” *and* “*unclear expectations*,” as well as “*excessive workload*,” were cited as additional stressors, along with “*too high nurse-patient ratios*” and a “*dysfunctional workplace environment*.” Despite facing a large number of patients, many participants felt “*unheard*.” Furthermore, they articulated their complaints and stories, affirming, “*No one listened to our complaints/stories*.”

##### Individual level

Reflecting on the impact of burnout among nurses on an individual level, participants shared a variety of personal experiences. Many spoke of feeling “*overwhelmed by work*” and “*working without feelings or empathy*,” highlighting the emotional toll of burnout. Some expressed discontent with “*unfair patient assignments*” and the burden of “*additional non-clinical tasks*,” which intensified their stress. “*Managing difficult patients*” and “*handling increased workload without compensation*” further added to their strain. Participants highlighted “*inflexible duty schedules*” and the difficulties of “*changing tasks*,” “*environments*,” and “*roles*” as contributors to their burnout. The pressures from management and the absence of adequate breaks, such as “*short vacations*” and “*insufficient break time*,” emphasized the relentless nature of their workload. Interpersonal relationships also contributed, including “*disconnection from the team*,” “*unsupportive managers*,” and a “*persistent feeling of being unvalued*.” Moreover, problems like “*low salaries*,” “*unfair treatment*,” and “*toxic workplaces*” significantly affected the participants, impacting their overall well-being. The stress of “*overtime*,” “*conflicting roles*,” and “*poor communication*” further intensified feelings of burnout, leading to a sense of isolation and frustration among nurses.

##### Interpersonal level

In exploring the impact of burnout among nurses at the interpersonal level, participants shed light on numerous effects. Many described experiencing “*stress*” and “*anxiety*,” which led to “*limited movements and personal interactions*” within their professional and personal spheres. “*Fatigue*,” “*loss of energy*,” and “*emotional and physical exhaustion*” were common sentiments, with one participant expressing “*emotional exhaustion*.” Some nurses noted difficulties coping with stressors, mentioning “*exaggerating small concerns*” and “l*ack of adaptation between day and night shifts*.” The toll of burnout manifests in various ways, including “*loss of energy*,” “*depletion*,* fatigue*,” “*overexertion*,” “*withdrawal*,” “*increased mental distance from one’s job*,” “*feelings of negativity*,” and “*reduced sense of personal productivity*.” Others described feelings of “*overreacting*,” “*overanalyzing*,” and “*not speaking up*,” highlighting the strain experienced. Participants emphasized the burden of pressures and challenges of maintaining a professional demeanor, noting “*mannerisms*” as a coping mechanism. Moreover, burnout resulted in a “*reduced sense of personal accomplishment*,” “*feeling isolated*,” and “*lack of sleep*.” Additional stressors such as “*language barriers*,” “*gossip*,” and “*problems with work-life balance*” were identified, further contributing to the sense of burnout and isolation. Despite these challenges, participants highlighted the importance of “*leadership support*” in alleviating burnout.

### Theme 2–Implications of burnout

#### This thematic construct aimed to evaluate the participants’ Understanding of the implications of burnout at

##### Organization level

The implications of nurses’ burnout resonate across various aspects of healthcare delivery. Participants voiced concerns about the potential for “*ineffective care delivery*” and a “*high turnover rate*” in their workplaces, emphasizing how burnout can adversely impact the quality of care. The repeated emphasis on “*quality of care*” underscores its significance in discussions about burnout as nurses face the consequences of their emotional and physical exhaustion. Instances of nurses resigning from their roles further exacerbate staffing shortages and create a cycle of turnover, ultimately threatening patient safety and the overall quality of healthcare delivery. Moreover, burnout results in “*decreased productivity*” and “*negligence*,” presenting considerable risks to patient well-being. The consequences extend to patient satisfaction levels, with reports of “*decreased patient satisfaction*” illustrating the extensive impact of burnout on the healthcare experience. The increase in “*increased sick leave*” among burned-out nurses further burdens organizational resources and accentuates the urgency of addressing burnout at its source. A common perception of a “*poor work environment*” heightens these challenges, fostering disengagement among staff and perpetuating a cycle of dissatisfaction.

##### Individual level

The prevalence of burnout among nurses has several harmful effects, significantly impacting their professional effectiveness and personal well-being. Nurses experiencing burnout often find their job performance affected, facing an “*increased risk of leaving the workplace.*” The frequency of mistakes, including “*medication errors*,” is worsened by feelings of a “*lack of support*” and “*work overload*.” Strained relationships among colleagues contribute further to “*increased errors*” and “*conflicts*,” while diminishing workplace effectiveness. This cycle of reduced performance is coupled with a persistent sense of dissatisfaction, characterized by “*low motivation*” and “*decreased staff satisfaction*.” Burnout also disrupts social interactions and teamwork, creating a “*negative work environment*” marked by “*tardiness*,” “*neglect of work*,” and a “*preference for other employees*.”

##### Interpersonal level

Nurses affected by burnout endure adverse effects such as “*low self-esteem*” and “*health-related issues*.” “*Chronic overexertion*” undermines confidence in patient care and compromises quality. “*Mental instabilities*” and “*exhaustion*” increase frustration, obstructing communication and collaboration. Nurses struggle with “*little patience*,” confront “*depression*,” “*physical impairment*,” and challenges in providing “*compassionate care*.” Feeling “*exhausted at all times*” fosters negativity, underscoring the need for support systems to maintain well-being and the integrity of patient care.

### Theme 3–Proposed/Suggested coping mechanisms

#### This thematic construct aimed to assess the individual self-proposed coping mechanisms

##### Personal/self-awareness and self-monitoring

In response to the challenges posed by burnout, nurses have proposed various coping mechanisms. They emphasized the importance of managing time wisely and engaging in “*walking*” *and* “*reading*” to promote self-awareness and self-care. “*Meditation*,” “*hobbies*,” and “*open communication with family*” were also highlighted as effective strategies for maintaining mental well-being. Nurses stressed the need to continuously “*improve themselves*” and “*monitor themselves*” to prevent burnout. Additionally, creating a supportive work environment through “*working hard and helping each other*” was emphasized. Some have suggested practical measures such as “*reducing working hours*,” “*seeking external support from family*,” or considering “*alternative career paths*.” “*Rest*,” “*relaxation*,” and “*engaging in enjoyable activities*” were advocated as essential for rejuvenation and resilience-building. Cultivating positivity through “*expressing feelings to a friend*” and “*acknowledging burnout triggers*” were vital strategies. Ultimately, nurses emphasized the importance of “*self-discipline*,” “*setting boundaries*,” and “*finding purpose in their work*” to navigate the demands of their profession while preserving their well-being.

##### Workplace environment

To mitigate burnout and cultivate a supportive workplace environment, nurses have proposed several actionable strategies, echoing participants’ quotations. They advocate for the creation of a “*comfortable workplace*” alongside fostering a culture of “*helping each other*” and prioritizing “*communication and respect among coworkers*.” “*Team building*” initiatives are emphasized, as is the importance of “*avoiding misbehavior in front of patients or colleagues*.” Suggestions such as instituting “*monthly recognition for the best nurse*” and implementing “*better scheduling*” aim to boost morale and job satisfaction. Addressing issues promptly during working hours and providing a “*stress-free environment*” are crucial for maintaining staff well-being. Finally, “*approachable leadership*” is pivotal in nurturing a positive workplace culture.

##### Nursing administration responsibilities

Prioritizing the integration of “*new staff*” into the team and “*recognizing and appreciating deserving individuals*” are crucial strategies. Suggestions include granting sufficient “*vacation* time” and providing opportunities for “*off-days or holidays*” to promote work-life balance. “*Decrease overtime and extend vacation time*” aims to alleviate workload pressures and motivate staff. Additionally, “*distributing tasks equally*,” “*offering scheduling flexibility*,” “*ensuring adequate break time*,” *and* “*fair assignments* are essential for managing workload and promoting staff well-being. “*Listening to staff complaints*” and “*addressing concerns*” promptly foster a supportive culture. Granting sufficient time off to restore both physical and mental health, as stated by one participant: “*Staff who seem unmotivated at work need to have at least one day off to regain energy and mental state.*”

##### Organization responsibilities

Emphasizing the importance of “*sick leave when needed*” ensures staff well-being and reduces stress levels. Participants advocated for “*increasing the number of nursing staff*” to alleviate workload burdens and enhance the quality of patient care. Incorporating “*recreational and motivational activities*” to boost staff morale and foster engagement. Suggestions such as an “*increase in salary*” and “*providing adequate compensation*” highlight the importance of recognizing and rewarding staff contributions. Implementing an “*annual staff satisfaction survey*,” as mentioned by one participant, allows for feedback collection and organizational improvement. Furthermore, another participant noted that “*assessing the value of service provided to patients*” underscores the significance of staff dedication. Strategies such as “*reducing working hours*,” “*granting more leave or off-duty time*,” and “*providing psychological support*” are essential for combating burnout. Additionally, “*hiring more staff*” and “*depositing overtime pay on time*” contribute to staff satisfaction and well-being.

## Discussion

This study aimed to investigate nurses’ knowledge and awareness of the impact of workload and the roles nurses and hospital leaders play in overcoming burnout. It also explored the consequences of burnout on personal, individual, and organizational levels and the nurses’ self-coping techniques to mitigate it.

Nurses predominantly provided favorable feedback and urged all suggested ways to mitigate burnout triggers. Previous literature supported the study findings by emphasizing the significant role of nurse leaders in enhancing a positive work culture by fostering open communication, listening actively, and providing emotional support to reduce the triggers of burnout [51, 52]. Nurses advised their leaders to improve work conditions and empower them to mitigate symptoms of burnout [53], foster autonomy, and engage them in choices about patient care as a means to control workplace-related burnout triggers [54]. They urged their leaders to implement particular frameworks and work circumstances, including flexible scheduling, professional development opportunities, and supportive team settings that empower them to exercise professional autonomy [55], to achieve a balance between their work and social lives.

Workload factors are mainly tied to nurse leadership. This study found that nurses recommended reducing workload factors by lowering the nurse-to-patient ratio, which reduced burnout, turnover rates, quality of nursing care, and patient satisfaction [56, 57]. Additionally, promoting work-life balance and creating support systems, including mentorship programs, enable flexible scheduling and sufficient leave days [58]. Nurses, particularly novices, may possess clinical proficiency yet lack essential nonclinical skills that could impact their performance and well-being, rendering them susceptible to negative workplace outcomes; thus, mitigating nonclinical responsibilities would be an effective strategy to alleviate workloads and reduce burnout triggers [59]. Previous studies emphasized that failure to control workload factors led to elevated levels of personal burnout, client-related burnout, job dissatisfaction, and a greater intention to leave the current position [60]. Consequently, an improved work environment and diminished perceived workload were consistently linked to a decreased risk of all adverse outcomes [61].

The National Academies of Sciences, Engineering, and Medicine (NASEM) has urged organizations to formulate, execute, and assess individual burnout interventions [62]. Nurses’ responses corroborated the findings of the prior studies by highlighting the crucial roles of hospital leaders in proactively addressing the burnout triggers through the implementation of prevention programs, mental health education, and resilience training to enhance employee mental health, patient care, and outcomes [63]; and by assisting them in utilizing their self-coping mechanisms to mitigate burnout symptoms, physical health, enthusiasm for their work, and realistic expectations [64, 65]. The study results aligned with recent literature, indicating that the relationship between sickness presenteeism and health-related productivity loss was partially linked to job burnout. Therefore, hospital and nurse managers can reduce the detrimental impact of sickness presenteeism on health-related productivity loss by addressing job fatigue, enhancing social support, and tackling these substantial problems; they can more efficiently mitigate the effects of sickness presenteeism and occupational burnout among nurses [66].

The findings of this study are corroborated by prior research, indicating that nurses who experienced burnout exhibited different responses compared to those who did not. This discrepancy is ascribed to the psychological environment fostered by leaders and the self-developed coping mechanisms, which significantly adversely impact new-experience burnout and turnover intention [67]. Nurses engaged in mentorship programs exhibited a markedly lower incidence of burnout compared to their counterparts who did not participate [68, 69].

The majority of participants were female, indicating the predominance of women in the nursing profession [70]. This is a concerning indicator, as evidence indicates that women report significantly higher levels of burnout than men, leading to more frequent exits from the workforce due to gender discrimination, biases, postponed personal life choices, and obstacles to career progression [71].

Age was identified as a predictor of reduced burnout and increased engagement via the application of surface-acting and anticipatory deep-acting emotion control methods [72]. Young employees were vulnerable to workplace demands and the lack of particular job resources, while older employees in managerial positions showed enhanced resilience [73]. Age and experience had a negative correlation with emotional weariness and depersonalization, while demonstrating a positive correlation with personal accomplishment [74]. Consequently, it is essential to implement interventions that mitigate burnout and address age-related strengths and vulnerabilities in job capacity [75]. Individuals aged 20–35 years and those over 55 years are susceptible and should be prioritized in efforts to mitigate burnout triggers [76, 77].

There is a high occurrence of sexist and racial microaggressions against females and racial minorities, which are linked to burnout [78]. Feelings of being incompetent despite experiencing successes were significantly associated with ethnicity and burnout [79].

The study findings showed that the nursing practice environment and nurse job outcomes were superior in military hospitals than in public hospitals, and the patient-to-nurse ratio was identified as a predictor of burnout and job dissatisfaction [80]. Private hospital nurses exhibited higher levels of emotional exhaustion and depersonalization than did public hospital nurses [81]. In contrast, Palestinian nurses working in governmental hospitals exhibited the highest levels of EE and DP and the lowest levels of PA compared to nurses who were working in private hospitals [82]. A moderate level of burnout was identified among nurses working in a university hospital [83].

Evidence has suggested that burnout symptoms are prevalent among nurses in various specializations and countries [84]; therefore, regardless of the hospital setting, burnout nurses exhibit high levels of burnout, high levels of EE and DP, and low PA [85, 86]; however, nurses working in tertiary centers are at the greatest risk of developing higher levels of burnout and secondary traumatic stress scores than those working in other centers [87, 88].

This study indicates that nurses on night shifts are more susceptible to burnout, validating the elevated prevalence of depression and poor sleep quality, which adversely affect patient safety and performance; nonetheless, numerous nurses on day shifts report markedly higher levels of burnout syndrome. Prolonged shifts and daytime dysfunction were significantly associated with burnout [89]. Rotating or irregular shifts were an additional factor associated with increased burnout rates and work dissatisfaction [90]. A comparison of 12-hour shifts (day versus night) indicated that night-shift nurses encounter more significant challenges with performance, and their fatigue was particularly pronounced after a 12-hour shift [91]. A particular change in work is a determinant that affects compassion fatigue, compassion satisfaction, and burnout [92]. Furthermore, nocturnal employment is markedly correlated with the health-related quality of life of nurses [93].

Nurses emphasized that supportive leadership is essential for enhancing their job satisfaction, organizational commitment, and retention while simultaneously diminishing emotional weariness and burnout to sustain an empowered and engaged workforce [94, 95]. Optimistic transformational and authentic leadership styles have successfully inspired and motivated them while diminishing burnout triggers [96]. However, abusive and laissez-faire leadership negatively affects psychological well-being and diminishes job satisfaction [97].

This study revealed that the experience of burnout was significantly correlated with excessive workloads [98], ambiguous workload and role expectations [99], extended working hours [100], insufficient staffing [101], elevated nurse-patient ratios [102, 103], an increased number of patients [104], a dysfunctional workplace environment [105], non-monetary rewards [106], lack of appreciation [107], low salaries [108], inadequate compensation [109], and stressful work experiences stemming from bullying, familial stress, and public misunderstandings [110], as well as the COVID-19 pandemic, which were identified as significant triggering factors [111]. Additional triggering factors encompass disputes with other healthcare professionals and interactions with patients or their families [112]. Inadequate management support, insufficient staffing, erratic staffing alterations, and variable patient requirements are significant stresses and catalysts for burnout that impede the delivery of competent care due to their psychological impact [113]. Moreover, inequitable treatment [114], nurses perceiving their concerns as disregarded or expressing their grievances [115], less empathy or a lack of emotional connection with colleagues or patients are indicative of the emotional repercussions of burnout [116, 117]. Several participants indicated rigid duty schedules [118], alterations in their responsibilities, allocation of non-nursing or non-professional activities [119], experiences of role ambiguity or conflicts [120], insufficient or brief break periods [121, 122], and limited holiday durations [123]. In addition to prevalent predisposing factors such as stress, worry, weariness, and diminished energy [124], additional issues have been identified, including language obstacles [125, 126], gossip [127, 128], sentiments of negativism [129], and challenges related to work-life balance [130].

The study’s findings indicated that burnout adversely affects the quality of care [131], the efficiency of care delivery [132], the incidence of errors [133, 134], the safety of the work environment [135], and the intention to resign from the workplace [35]. Participants frequently encounter diminished personal productivity and depersonalization at an individual level [136]. Additionally, certain participants encountered repeated sick leave [137] and diminished pleasure [138], while others had interrupted sociability [139] and teamwork [140].

This study revealed that nurses who recounted their detrimental burnout experiences and associated psychological impacts proposed helpful coping methods to mitigate and overcome this severe condition. According to the nurses, essential activities for rejuvenation and resilience include walking and reading [141]; mindfulness meditations [142]; communication and support from family [143]; self-monitoring and self-improvement [144]; fostering a healthy and constructive work environment [145]; rest, relaxation, and engaging in pleasurable activities [146, 147]; recognizing and addressing burnout [148, 149]; as well as self-discipline and establishing boundaries [150, 151]. These elements significantly contribute to the enhancement of self-awareness and self-care. The suggested workplace mechanisms, including the establishment of a stress-free environment [125], the prevention of misconduct/incivility or workplace adversity [152, 153], enhanced job involvement, a flexible work setting [154, 155], accessible leadership [156, 157], and significant recognition [158], along with workplace motivation [159], and the effective integration of new nurses through a comprehensive orientation [160, 161] are highly advocated for mitigating burnout among nursing personnel. Some participants believed that improving job satisfaction [162], offering sufficient monetary compensation [163, 164], or ensuring timely payments might alleviate burnout [165].

## Conclusion

Workplace burnout is prevalent, especially among nurses. Many nurses face challenges in maintaining a balanced state, resorting to coping strategies to avoid stressors, and seeking ways to alleviate their burnout, which in turn leads to negative impacts on individual, personal, and organizational levels. Therefore, nurse leaders, along with workload strategies and hospital administrators, play a crucial role in mitigating and overcoming burnout. Establishing a healthy work environment is recognized as the most effective strategy for combating burnout, followed by implementing mental health education and training programs to enhance adaptive and cognitive resilience, promote health improvement, and strengthen resistance to burnout. Further research is necessary to assess the effectiveness of these coping strategies for other healthcare professionals and to examine how cultural diversity, religious beliefs, and social factors may affect burnout triggers, consequences, and the development of self-coping mechanisms.

### Implications for practice

#### Nurse leaders

Policymakers should amend the institutional policies to maintain appropriate staffing ratios to decrease workload stress, introduce equitable scheduling practices to enhance work-life balance, and enforce consistent, uninterrupted breaks to support mental and physical rejuvenation. Clear career progression routes and employee assistance programs for managing work-related stress are essential. Providing clear pathways for professional advancement and offering employee assistance programs for coping with job-related stress are crucial.

#### Hospital leaders

Organizations must enforce policies that directly confront the elements contributing to burnout. Organizational healthcare leaders should be vigilant and stay focused on improving resilience in hospital settings. They should consider nurses’ perceptions and create a supportive community among nurses to foster a team-oriented environment, emotional support, and reduced feelings of isolation.

### Recommendations

Combing our study findings with the latest evidence following a literature review, we recommend the following practical guides to assist leaders and organizations in addressing and preventing burnout: (1) offer stress management strategies, (2) enable employees to shape their work actively, (3) foster and promote social support, (4) involve employees in decision-making processes, (5) implement effective performance management, and (6) follow the socio-ecological model to prevent burnout.

### Strengths and limitations

This study showed that nurses’ views on burnout differ based on various predisposing factors, including age, ethnicity/race, type of hospital, level of hospital, type of work, type of shift, leadership style, previous burnout experience, proper orientation, years of nursing experience, and years of hospital experience. Nursing leaders and hospital administrators should work together to assess and address these factors proactively. Nurses from diverse cultural backgrounds have varying perceptions of burnout. Therefore, nursing leaders must be mindful of these differences and offer culturally congruent training and education. Previous studies have yet to look at the hospital’s level (e.g., tertiary or secondary).

This study has several limitations. First, it is limited to nurses working in the civil healthcare sector, with no involvement of other healthcare professionals or nurses working in the military healthcare sector. Second, possibilities of biased responses due to workplace pressures (e.g., Royal Hospital), held position, cultural diversity, self-perception of burnout, leadership, and management impacts would be expected. Third, the cross-sectional design precludes causation.

## Electronic supplementary material

Below is the link to the electronic supplementary material.

Supplementary Material 1

Supplementary Material 2

## Data Availability

No datasets were generated or analysed during the current study.

## Declarations

### Declaration of helsinki

This research falls under the category of “exempt,” and all authors possess active Good Clinical Practice certificates to ensure adherence to the Declaration of Helsinki during the study’s conduct. Additionally, before participants were enrolled, the researcher explained the study’s purpose and that participation was entirely voluntary. Furthermore, all participants were informed about the issues of anonymity and confidentiality, as well as the option to terminate the study at any time without affecting their current or future work.

## Abbreviations

WHO: World Health Organization
EE: Emotional Exhaustion
DP: Depersonalization
PI: Professional Inefficacy
PA: Personal Accomplishment
NR: Nursing Administration Responsibilities
HR: Hospital Administration Responsibilities
WL: Workload
NASEM: National Academies of Sciences, Engineering, and Medicine
RN: Registered Nurse
SN: Staff Nurse
CN: Charge Nurse
SM: Shift Manager
HN: Head Nurse
UM: Unit Manager
NM: Nurse Manager
ND: Nurse Director
CI: Clinical Instructor
NE: Nurse Educator
SNE: Senior Nurse Educator
ICU: Intensive Care Unit
CCU: Coronary Care Unit
OT: Operation Theatre
OR: Operating Rooms
PACU: Post Anesthesia Care Unit
RDU: Renal Dialysis Unit
DCU: Day Care Unit
PHC: Primary Care Center
KFMC: King Fahad Medical City
KSMC: King Saud Medical City
KFSH&RC: King Faisal Specialist Hospital and Research Center)
PMAH: Prince Mohammed Bin Abdulaziz Hospital
HMG: Al Habib Medical Group HMG
