# Supplementary material for: Burnout combating strategies, triggers, implications, and self-coping mechanisms among nurses working in Saudi Arabia: a multicenter, mixed methods study
Source: BMC Nurs. 2025 May 26;24:590. doi: 10.1186/s12912-025-03191-w (PMC12107983; doi:10.1186/s12912-025-03191-w)
Supplement: Supplementary file 1 — Supplementary Material 1 [file 12912_2025_3191_MOESM1_ESM.pdf]

Dear Participants,

Greetings!

We invite you to participate in a survey entitled **Burnout triggering factors, implications, coping mechanisms, and combating strategies among nurses working in Saudi Arabia: A multicenter, mixed methods study**. The study aims to identify strategies to combat burnout among nurses in hospitals and other healthcare settings, assess the contributing factors, and compare the nurses' views in different settings and at different levels.

Your participation in this survey is optional, and the privacy of the information provided will be guaranteed. The answers are confidential, and the study results will be analyzed and reported statistically as a group. Your responses cannot be identified.

Data from this survey will be kept confidential and reported only as a collective total. Only the surveyors will know your answers to this questionnaire.

If you agree to participate in this survey project, please answer the questions on the questionnaire as best you can. It should take approximately *15 minutes* to complete.

Don't hesitate to contact the surveyors if you have any questions about this research project. Thank you for your assistance in this important endeavor. If you need to enquire about this survey, kindly contact:

Name: Mohammad Jaber
E-mail: jaber71@uwindsor.ca

Sincerely Yours,

Principal Investigator

№	**Part 1: Sociodemographic Data**					
1.	Gender:	☐ Male	☐ Female			
2.	Age:	☐ ≤ 25	☐ 26 – 30	☐ 31 – 35	☐ 36 – 40	☐ > 40
3.	Marital Status:	☐ Single	☐ Married	☐ Widow	☐ Divorced	☐ Separated
4.	Ethnicity/Race:	☐ Middle East	☐ Asia	☐ Africa	☐ America	☐ Europe
		☐ India	☐ Hispanic/Latino			
5.	Education Level:	☐ Diploma	☐ Bachelor	☐ Master	☐ Doctorate	
6.	Professional Title:	☐ SN (RN)	☐ CN (SM)	☐ HN (UM)	☐ NM	☐ ND
		☐ CI	☐ NE	☐ SNE	☐ Chairperson/Chairman	
7.	Type of Hospital:	☐ Governmental	☐ General	☐ Educational	☐ Private	
		☐ Public Health Centers				
8.	Level of Hospital:	☐ Tertiary	☐ Secondary	☐ Primary	☐ Clinic	
9.	Area of Work:	☐ Critical Area (e.g., ICU/CCU/Burn)		☐ OT (e.g., OR/Cath Lab/PACU)		☐ EDs
		☐ Inpatient Wards	☐ Outpatient Clinic	☐ Ambulatory (e.g., RDU/DCU/Endoscopy)		
		☐ PHC	☐ Other			
10.	Type of Work:	☐ Administrative	☐ Clinical	☐ Education	☐ Quality	☐ Other
11.	Type of Shift:	☐ Morning (8-9-hrs)	☐ Day (12-hr)	☐ Night (12-hr)	☐ Rotating Shift (12-hr)	
		☐ Rotating Shift (8-9-hrs)				
12.	Weekly Working Hours:		☐ ≤ 45 hrs	☐ 46 – 48 hrs	☐ > 48 hrs	
13.	Nursing Care Delivery Method:					
		☐ Total Patient Care	☐ Team	☐ Functional	☐ Unclear	
14.	Leadership Style:	☐ Autocratic	☐ Democratic	☐ Transactional	☐ Transformational	
		☐ Permissive/Laissez-faire				
15.	Years of Experience in Nursing:					
		☐ < 1	☐ 1 – 5	☐ 6 – 10	☐ 11 – 15	☐ > 15
16.	Years of Experience in the Hospital:					
		☐ < 1	☐ 1 – 5	☐ 6 – 10	☐ 11 – 15	☐ > 15
17.	Did you experience burnout?					
		☐ Yes	☐ No			
18.	Did you receive training related to managing burnout?					
		☐ Yes	☐ No			
19.	Did you receive a proper orientation (preceptorship/mentorship) upon arrival at the unit?					
		☐ Yes	☐ No			

Legends: *RN: Registered Nurse, SN: Staff Nurse, CN: Charge Nurse, SM: Shift Manager, HN: Head Nurse, UM: Unit Manager, NM: Nurse Manager, ND: Nurse Director, CI: Clinical Instructor, NE: Nurse Educator, SNE: Senior Nurse Educator, ICU: Intensive Care Unit, CCU: Coronary Care Unit, OT: Operation Theatre, OR: Operating Rooms, PACU: Post Anesthesia Care Unit, RDU: Renal Dialysis Unit, DCU: Day Care Unit, PHC: Primary Care Center.*

Section A: Quantitative Study

Part 1: Nursing Administration Responsibilities (NR)

- This part includes 12 items that evaluate the "Responsibilities of Nursing Managers & Leaders."
- This part uses a 3-Point Scale to assess the role of nurse managers and leaders in assessing and managing nurses' burnout:
  - *Leaders' Training to Recognize, Address, and Manage Burnout* subscale has six items concerned with the ability of nurse managers and leaders to assess their staff experiencing burnout and provide appropriate management.
  - *Nurses' Engagement in Unit Businesses and Scheduling* subscale has six items that assess the impact of reinforcing the nursing staff's professional accountability and autonomy and the outcomes of nurses' self-scheduling on their emotional/psychological, mental, and personal experience.

№	Statements	Criteria		
		Disagree (1)	Neutral (2)	Agree (3)
Leaders' Training to recognize, address, and manage Burnout:				
1	Nurse leaders should be familiar with the signs of a nurse who may be disengaged or experiencing burnout.			
2	Nurse leaders should take steps to support their staff and find ways to address stress levels before burnout becomes a more serious issue.			
3	Nurse leaders should identify the factors leading to burnout, e.g., work shift, emotional exhaustion (e.g., loss of energy, depletion, overextension, fatigue), depersonalization (withdrawal, increased mental distance from one's job, feeling of negativism/cynicism related to one's job), and professional Inefficacy/Personal Accomplishment (reduced feelings of personal accomplishment or productivity).			
4	Nurse leaders should improve the work environment and conditions.			
5	Nurse leaders should empower and motivate nurses.			
6	Nurse leaders should initiate interventions that focus on the meaning of job satisfaction, improve nurses' perceptions about their jobs, and generate benefits, as well as professional and career growth.			
Nurses' Engagement in Unit Businesses and Scheduling:				
7	Nurse leaders should allow nurses to participate in decision-making related to their work.			
8	Nurse leaders should provide nurses with autonomy and control over their practice.			
9	Nurse leaders should reinforce the concept of shared governance and professional accountability among nurses to sustain strong nursing work.			
10	Nurse leaders should involve nurses in leadership roles to improve the quality of the nursing work environment and decision-making.			
11	Allowing nurses to have more control of their schedule would reduce fatigue, emotional exhaustion, and depersonalization.			
12	Allowing nurses to have more control over their schedule would lead to a better work/life balance.			

Part 2: Impact of Workload (WL)

- This part includes 12 items that evaluate the "Impact of Workload."
- This part uses a 3-Point Scale to assess the nurses' attitudes or opinions (views & perceptions) about the use of proposed strategies to combat burnout:
  - *Adjusting Nurse-to-Patient Ratios and Working Hours* subscale has nine items concerned with the association between the number of patients assigned to each nurse and the quality of nursing care, nurses' turnover rate, readmission rate, and patients' satisfaction.
  - *Reducing Non-Clinical Tasks* subscale has three items evaluating the effect of reducing non-clinical work on nurses' quality of care and patient outcomes.

№	Statements	Criteria		
		Disagree (1)	Neutral (2)	Agree (3)
Adjustment of Nurse-to-Patient Ratios and Working Hours:				
13	Adjusting the nurse-to-patient ratio is more likely to reduce emotional exhaustion, job dissatisfaction, and burnout.			
14	Adjusting the nurse-to-patient ratio may require additional staff, but the expense can offset other challenges.			
15	Adjusting the nurse-to-patient ratio reduces nurse turnover.			
16	Adjusting the nurse-to-patient ratio improves the quality and outcomes of healthcare.			
17	Adjusting the nurse-to-patient ratio improves patient satisfaction.			
18	Adjusting the nurse-to-patient ratio may reduce readmission rates.			
19	Each additional patient per nurse is associated with an increase in the mortality rate, e.g., an increase in the likelihood of failure to rescue and the likelihood of dying within 30 days of admission.			
20	Using external nurses to fill vacant positions rather than relying on local nurses to fill all available positions helps reduce work fatigue and prevent burnout.			
21	Adjusting working hours by reducing overtime and avoiding long shifts reduces work fatigue, maintains patient safety, minimizes adverse events/incidents (medication errors, infections, patient falls), improves the quality of care and job performance, and reduces the intention to leave (turnover rate).			
Reducing Non-Clinical Tasks:				
22	Reducing non-clinical work keeps nurses focused on valuable clinical tasks.			
23	Reducing non-clinical work keeps nurses from being depressed and reduces their workload, prevents distractions, and maintains mental health.			
24	Assigning non-clinical tasks such as post-discharge follow-up, monitoring, and visit scheduling to other workers keeps nurses more engaged in patient care, improves patient experience, and improves healthcare outcomes.			

<u>Part 3: Hospital Administration Responsibilities (HR)</u>

- This part includes 12 items that evaluate the "Responsibilities of the Hospital Administration."
- This part uses a 3-point Scale to assess the role of hospital administration in preventing burnout and creating a healthy workplace.
  - *The regular Workday and Physical Breaks* subscale has six items that evaluate the effect of regular recovery time and social interaction activities in overcoming burnout and burnout-related factors.
  - *The implementing Support Programs* subscale has six items that evaluate the importance of resilience training programs and incentive exercises in combating burnout and burnout-triggering factors.

**№**	**Statements**	**Criteria**		
		Disagree (1)	Neutral (2)	Agree (3)
Regular Workday and Physical Breaks:				
25	Teaching nurses better break scheduling helps them to cope with work pressures.			
26	Holding departmental meetings to talk about health helps nurses to release stressors and work pressures.			
27	Creating comfortable respite areas for nurses reduces work stressors and prevents burnout.			
28	Assessing the nurses' needs and listening to feedback helps them cope and prevent burnout.			
29	Developing teamwork and improvement courses helps to minimize labor stress.			
30	Teaching individual nurses how to separate work from home life prevents burnout.			
Implementing Support Programs:				
31	Creating wellness teams can keep health front and center for nurses and promote mental, emotional, spiritual, and social opportunities.			
32	Cognitive coping strategies, cognitive-behavioral interventions, and cognitive evaluation reduce stress reactions at work.			
33	Initiating mindfulness-based courses, mental attention training, spiritual pain assessment, and psychological empowerment programs reduces emotional exhaustion and burnout symptoms.			
34	Initiating communication skill training and professional identity development programs reduces burnout.			
35	Initiating meditation improves relaxation and life satisfaction, improves mental health, and enhances a healthy lifestyle.			
36	Reinforcing the 'zero violence/behavioral standards policy' that maintains nurses' respect, prevents job harassment and bullying, and reduces burnout and turnover.			

Section B: Qualitative Study

**Part 1: Perceived Knowledge of Burnout and Coping Mechanisms**<br>• This part includes three themes evaluating the individual perception of burnout contributing factors, implications, and preferred personal coping strategies.<br>1. Theme 1: This thematic construct aims to assess the ability of the individual nurse knowledge to identify the contributing factors that trigger burnout symptoms about Emotional Exhaustion (EE) (e.g., loss of energy, depletion, overextension, fatigue), Depersonalization (DP) (e.g., withdrawal, increased mental distance from one's job, feeling of negativism/cynicism related to one's job), Professional Inefficacy/Personal Accomplishment (PI/PA) (e.g., reduced feelings of personal accomplishment or productivity) at the:<br>▪ *Organization Level.*<br>▪ *Individual Level.*<br>▪ *Interpersonal Level.*<br>2. Theme 2: This thematic construct aims to assess the implications of burnout at the:<br>▪ *Organization Level.*<br>▪ *Individual Level.*<br>▪ *Interpersonal Level.*<br>3. Theme 3: This thematic construct aims to assess the individually proposed/suggested coping mechanisms about:<br>▪ *Personal/Self-Awareness & Self-Monitoring.*<br>▪ *Workplace Environment.*<br>▪ *Nursing Administration Responsibilities.*<br>▪ *Organization Responsibilities.*
**Theme 1:** Please describe the contributing factors that trigger burnout about EE, DP, and PI/PA at the Interpersonal/Individual and Organizational Levels.
**Theme 2:** Please describe the effects of burnout at the Interpersonal/Individual Level and Organization Level.
**Theme 3:** Please suggest your coping strategies regarding Personal/Self-Awareness and self-monitoring, Workplace Environment, Nursing Management Responsibilities, and Organizational Responsibilities.

End of The Questionnaire
