# Supplementary material for: Burnout combating strategies, triggers, implications, and self-coping mechanisms among nurses working in Saudi Arabia: a multicenter, mixed methods study
Source: BMC Nurs. 2025 May 26;24:590. doi: 10.1186/s12912-025-03191-w (PMC12107983; doi:10.1186/s12912-025-03191-w)
Supplement: Supplementary file 2 — Supplementary Material 2 [file 12912_2025_3191_MOESM2_ESM.docx]

**Burnout combating strategies, triggers, implications, and self-coping mechanisms among nurses working in Saudi Arabia: A multicenter, mixed methods study**

Tables-Supplementary (S1-S7)

| **Table S1:** *Spearman's rho* correlations | | | | | |
| --- | --- | --- | --- | --- | --- |
|  | | **Total Score** |  | | **Total Score** |
| Q1 | Correlation Coefficient | 0.491 | Q19 | Correlation Coefficient | 0.600 |
|  | Sig. (2-tailed) | **<.001** |  | Sig. (2-tailed) | **<.001** |
|  | N | 1747 |  | N | 1747 |
| Q2 | Correlation Coefficient | 0.51 | Q20 | Correlation Coefficient | 0.592 |
|  | Sig. (2-tailed) | **<.001** |  | Sig. (2-tailed) | **<.001** |
|  | N | 1747 |  | N | 1747 |
| Q3 | Correlation Coefficient | 0.518 | Q21 | Correlation Coefficient | 0.565 |
|  | Sig. (2-tailed) | **<.001** |  | Sig. (2-tailed) | **<.001** |
|  | N | 1747 |  | N | 1747 |
| Q4 | Correlation Coefficient | 0.507 | Q22 | Correlation Coefficient | 0.581 |
|  | Sig. (2-tailed) | **<.001** |  | Sig. (2-tailed) | **<.001** |
|  | N | 1747 |  | N | 1747 |
| Q5 | Correlation Coefficient | 0.493 | Q23 | Correlation Coefficient | 0.593 |
|  | Sig. (2-tailed) | **<.001** |  | Sig. (2-tailed) | **<.001** |
|  | N | 1747 |  | N | 1747 |
| Q6 | Correlation Coefficient | 0.511 | Q24 | Correlation Coefficient | 0.557 |
|  | Sig. (2-tailed) | **<.001** |  | Sig. (2-tailed) | **<.001** |
|  | N | 1747 |  | N | 1747 |
| Q7 | Correlation Coefficient | 0.529 | Q25 | Correlation Coefficient | 0.573 |
|  | Sig. (2-tailed) | **<.001** |  | Sig. (2-tailed) | **<.001** |
|  | N | 1747 |  | N | 1747 |
| Q8 | Correlation Coefficient | 0.562 | Q26 | Correlation Coefficient | 0.585 |
|  | Sig. (2-tailed) | **<.001** |  | Sig. (2-tailed) | **<.001** |
|  | N | 1747 |  | N | 1747 |
| Q9 | Correlation Coefficient | 0.557 | Q27 | Correlation Coefficient | 0.539 |
|  | Sig. (2-tailed) | **<.001** |  | Sig. (2-tailed) | **<.001** |
|  | N | 1747 |  | N | 1747 |
| Q10 | Correlation Coefficient | 0.543 | Q28 | Correlation Coefficient | 0.514 |
|  | Sig. (2-tailed) | **<.001** |  | Sig. (2-tailed) | **<.001** |
|  | N | 1747 |  | N | 1747 |
| Q11 | Correlation Coefficient | 0.565 | Q29 | Correlation Coefficient | 0.526 |
|  | Sig. (2-tailed) | **<.001** |  | Sig. (2-tailed) | **<.001** |
|  | N | 1747 |  | N | 1747 |
| Q12 | Correlation Coefficient | 0.543 | Q30 | Correlation Coefficient | 0.578 |
|  | Sig. (2-tailed) | **<.001** |  | Sig. (2-tailed) | **<.001** |
|  | N | 1747 |  | N | 1747 |
| Q13 | Correlation Coefficient | 0.556 | Q31 | Correlation Coefficient | 0.575 |
|  | Sig. (2-tailed) | **<.001** |  | Sig. (2-tailed) | **<.001** |
|  | N | 1747 |  | N | 1747 |
| Q14 | Correlation Coefficient | 0.572 | Q32 | Correlation Coefficient | 0.597 |
|  | Sig. (2-tailed) | **<.001** |  | Sig. (2-tailed) | **<.001** |
|  | N | 1747 |  | N | 1747 |
| Q15 | Correlation Coefficient | 0.598 | Q33 | Correlation Coefficient | 0.616 |
|  | Sig. (2-tailed) | **<.001** |  | Sig. (2-tailed) | **<.001** |
|  | N | 1747 |  | N | 1747 |
| Q16 | Correlation Coefficient | 0.54 | Q34 | Correlation Coefficient | 0.588 |
|  | Sig. (2-tailed) | **<.001** |  | Sig. (2-tailed) | **<.001** |
|  | N | 1747 |  | N | 1747 |
| Q17 | Correlation Coefficient | 0.519 | Q35 | Correlation Coefficient | 0.583 |
|  | Sig. (2-tailed) | **<.001** |  | Sig. (2-tailed) | **<.001** |
|  | N | 1747 |  | N | 1747 |
| Q18 | Correlation Coefficient | 0.582 | Q36 | Correlation Coefficient | 0.549 |
|  | Sig. (2-tailed) | **<.001** |  | Sig. (2-tailed) | **<.001** |
|  | N | 1747 |  | N | 1747 |
| Correlation is significant at the 0.01 level (2-tailed). | | | | | |

| **Table S2:** KMO and Bartlett's Test | | |
| --- | --- | --- |
| Kaiser-Meyer-Olkin Measure of Sampling Adequacy. | | .965 |
| Bartlett's Test of Sphericity | Approx. Chi-Square | 42294.169 |
|  | *d*f | 630 |
|  | Sig. | .000 |

| **Table S3:** Reliability Statistics | |
| --- | --- |
| Cronbach's Alpha | N of Items |
| .937 | 36 |

| **Table S4:** Test of Normality (*n* = 1747) | | | | | | |
| --- | --- | --- | --- | --- | --- | --- |
|  | **Kolmogorov-Smirnov^a,b^** | | | **Shapiro-Wilk** | | |
|  | **Statistic** | **df** | **Sig.** | **Statistic** | **df** | **Sig.** |
| Nursing Administration Responsibilities | .283 | 1747 | **.000** | .637 | 1747 | <.001 |
| Impact of Workload | .223 | 1747 | **<.001** | .770 | 1747 | <.001 |
| Hospital Administration Responsibilities | .307 | 1747 | **.000** | .645 | 1747 | <.001 |
| a. Lilliefors Significance Correction.  b. Sample size ≥ 50. | | | | | | |

| **Table S5:** Participants' characteristics (*n* = 1747) | |
| --- | --- |
| ***Variables*** | ***Number (%)*** |
| **Gender**  Male  Female | 287 (17.0)  1450 (83.0) |
| **Age (Years)**  ≤25  26-30  31-35  36-40  >40 | 191 (10.9)  418 (23.9)  487 (27.9)  352 (20.1)  299 (17.1) |
| **Marital Status**  Single  Married  Widow  Divorced  Separated | 768 (44.0)  915 (52.4)  12 (0.7)  30 (1.7)  22 (1.3) |
| **Ethnicity/Race**  Middle East  Asia  Africa  America  Europe  Hispanic/Latino  India | 406 (23.2)  884 (50.6)  78 (4.5)  4 (0.2)  36 (2.1)  158 (9.0)  181 (10.4) |
| **Educational Level**  Diploma  Bachelor  Master  Doctorate | 333 (19.1)  1305 (74.7)  100 (5.7)  9 (0.5) |
| **Professional Title**  Registered Nurse (Staff Nurse)  Charge Nurse (Shift Manager)  Head Nurse (Unit Manager)  Nurse Manager  Nursing Director  Clinical Instructor  Nurse Educator  Senior Nurse Educator  Chairperson/Chairman | 1482 (84.8)  131 (7.5)  46 (2.6)  19 (1.1)  14 (0.8)  31 (1.8)  15 (0.9)  1 (0.1)  8 (0.5) |
| **Years of Experience in Nursing**  <1  1-5  6-10  11-15  >15 | 113 (6.5)  424 (24.3)  448 (25.6)  381 (21.8)  381 (21.8) |
| **Years of Experience in the Hospital**  <1  1-5  6-10  11-15  >15 | 227 (15.9)  579 (33.1)  392 (22.5)  264 (15.1)  235 (13.5) |

| **Table S6:** Participants' work conditions (*n* = 1747) | |
| --- | --- |
| ***Variables*** | ***Number (%)*** |
| **Type of Hospital**  Governmental  General  Educational  Private  Public Health Centers | 1023 (58.6)  141 (8.1)  94 (5.4)  462 (26.4)  27 (1.5) |
| **Level of Hospital**  Tertiary  Secondary  Primary  Clinic | 1112 (63.7)  341 (19.5)  252 (14.4)  42 (2.4) |
| **Area of Work**  Critical Care Units  Operation Theater  EDs  Inpatient Wards  Outpatient Clinics  Ambulatory  Primary Healthcare Center  Other | 386 (22.1)  163 (9.3)  349 (20.0)  633 (36.2)  86 (4.9)  82 (4.7)  34 (1.9)  13 (0.7) |
| **Type of Work**  Administrative  Clinical  Education  Quality  Other | 70 (4.0)  1262 (72.2)  10 (0.6)  8 (0.5)  397 (22.7) |
| **Type of Shift**  Morning (8-9-hr)  Day (12-hr)  Night (12-hr)  Rotating shift (12-hr)  Rotating shift (8-9-hr) | 359 (20.5)  259 (14.8)  84 (4.8)  847 (48.5)  198 (11.3) |
| **Weekly Working Hours**  ≤45 hrs  46-48 hrs  >48 hrs | 231 (13.2)  836 (47.9)  680 (38.9) |
| **Nursing Care Delivery Method**  Total Patient Care  Team  Functional  Unclear | 1280 (73.3)  308 (17.6)  128 (7.3)  31 (1.8) |
| **Leadership Style**  Autocratic  Democratic  Transactional  Transformational  Permissive/Laissez-faire | 377 (21.6)  930 (53.3)  133 (7.6)  243 (13.9)  64 (3.7) |
| **Did you experience work-related burnout?**  Yes  No | 1108 (63.4)  639 (36.6) |
| **Did you receive training related to managing burnout?**  Yes  No | 405 (23.2)  1342 (76.8) |
| **Did you receive a proper orientation (preceptorship/mentorship) upon arrival at the unit?**  Yes  No | 1479 (84.7)  268 (15.3) |

| **Table S7:** Descriptive analysis of nurses' responses (*n* = 1747) | | | | | | |
| --- | --- | --- | --- | --- | --- | --- |
| **№** | **Items** | **Negative n (%)** | **Neutral n (%)** | **Positive n (%)** | **Median**  **(IQR)** | **Decision*** |
| **Nursing Administration Responsibilities:** 12 items that evaluate the Responsibilities of Nursing Managers and leaders. | |  |  |  |  |  |
| 1 | Nurse leaders should be familiar with the signs of a nurse who may be disengaged or experiencing burnout. | 35  (2.0) | 297  (17.0) | 1415  (81.0) | 3  (3-3) | High |
| 2 | Nurse leaders should take steps to support their staff and find ways to address stress levels before burnout becomes a more serious issue. | 28  (1.6) | 212  (12.1) | 1507  (86.3) | 3  (3-3) | High |
| 3 | Nurse leaders should identify the factors leading to burnout, e.g., work shift, emotional exhaustion (e.g., loss of energy, depletion, overextension, fatigue), depersonalization (withdrawal, increased mental distance from one's job, feeling of negativism/cynicism related to one's job), and professional Inefficacy/Personal Accomplishment (reduced feelings of personal accomplishment or productivity). | 37  (2.1) | 249  (14.3) | 1416  (83.6) | 3  (3-3) | High |
| 4 | Nurse leaders should improve the work environment and conditions. | 22  (1.3) | 210  (12.0) | **1515**  **(89.7)** | 3  (3-3) | High |
| 5 | Nurse leaders should empower and motivate nurses. | 20  (1.1) | 182  (10.4) | 1545  (88.4) | 3  (3-3) | High |
| 6 | Nurse leaders should initiate interventions that focus on the meaning of job satisfaction, improve nurses' perceptions about their jobs, and generate benefits, as well as professional and career growth. | 16  (0.9) | 210  (12.0) | 1521  (87.1) | 3  (3-3) | High |
| 7 | Nurse leaders should give nurses the opportunity to participate in decision-making related to their work. | 24  (1.4) | 239  (13.7) | 1484  (84.9) | 3  (3-3) | High |
| 8 | Nurse leaders should provide the nurses with autonomy and control over their practice. | 31  (1.8) | **357**  **(20.4)** | 1359  (77.8) | 3  (3-3) | High |
| 9 | Nurse leaders should reinforce the concept of shared governance and professional accountability among nurses to sustain strong nursing work. | 23  (1.3) | 265  (15.2) | 1459  (83.5) | 3  (3-3) | High |
| 10 | Nurse leaders should involve the nurses in leadership roles to improve the quality of the nursing work environment and decisional involvement. | 21  (1.2) | 247  (14.1) | 1479  (84.7) | 3  (3-3) | High |
| 11 | Allowing nurses to have more control of their schedule would reduce fatigue, emotional exhaustion, and depersonalization. | **59**  **(3.4)** | 336  (19.2) | 1352  (77.4) | 3  (3-3) | High |
| 12 | Allowing nurses to have more control of their schedule would have a better work/life balance. | 54  (3.1) | 336  (19.2) | 1352  (77.4) | 3  (3-3) | High |
| **Impact of Workload:** 12 items that evaluate the Impact of Workload. | |  |  |  |  |  |
| 13 | Adjusting the nurse-to-patient ratio is more likely to reduce the level of emotional exhaustion, job dissatisfaction, and burnout. | 50  (2.9) | 250  (14.3) | 1447  (82.8) | 3  (3-3) | High |
| 14 | Adjusting the nurse-to-patient ratio may require bringing in additional staff, but the expense can offset other challenges. | 69  (3.9) | 374  (21.4) | 1304  (74.6) | 3  (3-3) | High |
| 15 | Adjusting the nurse-to-patient ratio reduces nurse turnover. | 57  (3.3) | 321  (18.4) | 1369  (78.4) | 3  (3-3) | High |
| 16 | Adjusting the nurse-to-patient ratio improves the quality and the outcomes of healthcare. | 25  (1.4) | 226  (12.9) | 1496  (85.6) | 3  (3-3) | High |
| 17 | Adjusting the nurse-to-patient ratio improves patient satisfaction. | 21  (1.2) | 206  (11.8) | **1520**  **(87.0)** | 3  (3-3) | High |
| 18 | Adjusting the nurse-to-patient ratio may reduce readmission rates. | 97  (5.6) | 360  (20.6) | 1290  (73.8) | **3**  **(3-2)** | High |
| 19 | Each additional patient per nurse is associated with an increase in the mortality rate, e.g., an increase in the likelihood of failure to rescue and the likelihood of dying within 30 days of admission. | **119**  **(6.8)** | 458  (26.2) | 1170  (67.0) | **3**  **(3-2)** | High |
| 20 | Using external nurses to fill vacant positions rather than relying on local nurses to fill all available positions helps reduce work fatigue and prevent burnout. | 116  (6.6) | **492**  **(28.2)** | 1139  (65.2) | **3**  **(3-2)** | High |
| 21 | Adjusting working hours by reducing overtime and avoiding long shifts reduces work fatigue, maintains patient safety, minimizes adverse events/incidents (medication errors, infections, patient falls), improves the quality of care and job performance, and reduces the intention to leave (turnover rate). | 52  (3.0) | 318  (18.2) | 1377  (78.8) | 3  (3-3) | High |
| 22 | Reducing non-clinical work keeps nurses focused on valuable clinical tasks. | 33  (1.9) | 328  (18.8) | 1386  (79.3) | 3  (3-3) | High |
| 23 | Reducing non-clinical work keeps nurses depressurizes nurses' workload, prevents distractions, and maintains mental health. | 40  (2.3) | 305  (17.5) | 1402  (80.3) | 3  (3-3) | High |
| 24 | Assigning non-clinical tasks such as post-discharge follow-up, monitoring, and visit scheduling to other workers keeps nurses more engaged in patient care, improves patient experience, and improves healthcare outcomes. | 66  (3.8) | 342  (19.6) | 1339  (76.6) | 3  (3-3) | High |
| **Hospital Administration Responsibilities:** 12 items that evaluate the Responsibilities of The Hospital Administration. | |  |  |  |  |  |
| 25 | Teaching nurses better break scheduling helps them to cope with work pressures. | 55  (3.1) | 293  (16.8) | 1399  (80.1) | 3  (3-3) | High |
| 26 | Holding departmental meetings to talk about health helps nurses to release stressors and work pressures. | **59**  **(3.4)** | 307  (17.6) | 1381  (79.0) | 3  (3-3) | High |
| 27 | Creating comfortable respite areas for nurses reduces work stressors and prevents burnout. | 28  (1.6) | 241  (13.8) | 1478  (84.6) | 3  (3-3) | High |
| 28 | Assessing the nurses' needs and listening to their feedback helps them cope and prevent burnout. | 21  (1.2) | 191  (10.9) | **1535**  **(87.9)** | 3  (3-3)) | High |
| 29 | Developing teamwork and improvement courses helps to minimize labor stress. | 19  (1.1) | 227  (13.0) | 1501  (85.9) | 3  (3-3) | High |
| 30 | Teaching individual nurses how to separate work from home life prevents burnout. | 53  (3.0) | 283  (16.2) | 1411  (80.8) | 3  (3-3) | High |
| 31 | Creating wellness teams can keep health front of mind for nurses and promote mental, emotional, spiritual, and social opportunities. | 22  (1.3) | 297  (17.0) | 1428  (81.7) | 3  (3-3) | High |
| 32 | Initiating cognitive coping strategies, cognitive-behavioral interventions, and cognitive evaluation reduces stress reactions at work. | 26  (1.5) | 312  (17.9) | 1409  (80.7) | 3  (3-3) | High |
| 33 | Initiating mindfulness-based courses, mental attention training, spiritual pain assessment, and psychological empowerment programs reduce emotional exhaustion and burnout symptoms. | 39  (2.2) | **330**  **(18.9)** | 1378  (78.9) | 3  (3-3) | High |
| 34 | Initiating communication skill training and professional identity development programs reduces burnout. | 41  (2.3) | 296  (16.9) | 1410  (80.7) | 3  (3-3) | High |
| 35 | Initiating meditation improves relaxation and life satisfaction, improves mental health, and enhances a healthy lifestyle. | 48  (2.7) | 290  (16.6) | 1409  (80.7) | 3  (3-3) | High |
| 36 | Reinforcing the 'zero violence/behavioral standards policy' that maintains nurses' respect, prevents job harassment and bullying, and reduces burnout and turnover. | 19  (1.1) | 226  (12.9) | 1502  (86.0) | 3  (3-3) | High |
| *Decision-weighted mean rank average = 3 | | | | | | |
